# A Helical Polypeptide‐Based Potassium Ionophore Induces Endoplasmic Reticulum Stress‐Mediated Apoptosis by Perturbing Ion Homeostasis

**DOI:** 10.1002/advs.201801995

**Published:** 2019-05-24

**Authors:** DaeYong Lee, Soo‐Hwan Lee, Ilkoo Noh, Eonju Oh, Hyunil Ryu, JongHoon Ha, SeongDong Jeong, Jisang Yoo, Tae‐Joon Jeon, Chae‐Ok Yun, Yeu‐Chun Kim

**Affiliations:** ^1^ Department of Chemical and Biomolecular Engineering Korea Advanced Institute of Science and Technology (KAIST) Daejeon 34141 Republic of Korea; ^2^ Department of Bioengineering College of Engineering Hanyang University Seoul 04763 Republic of Korea; ^3^ Department of Biological Engineering Inha University Incheon 22212 Republic of Korea

**Keywords:** apoptosis, cancer therapy, ER stress, perturbed potassium homeostasis, potassium ionophore

## Abstract

Perturbation of potassium homeostasis can affect various cell functions and lead to the onset of programmed cell death. Although ionophores have been intensively used as an ion homeostasis disturber, the mechanisms of cell death are unclear and the bioapplicability is limited. In this study, helical polypeptide‐based potassium ionophores are developed to induce endoplasmic reticulum (ER) stress‐mediated apoptosis. The polypeptide‐based potassium ionophores disturb ion homeostasis and then induce prolonged ER stress in the cells. The ER stress results in oxidative environments that accelerate the activation of mitochondria‐dependent apoptosis. Moreover, ER stress‐mediated apoptosis is triggered in a tumor‐bearing mouse model that suppresses tumor proliferation. This study provides the first evidence showing that helical polypeptide‐based potassium ionophores trigger ER stress‐mediated apoptosis by perturbation of potassium homeostasis.

## 1 Introduction

A potassium electrochemical gradient is established by potassium channels that drive the transport of potassium cations by an active transport mechanism (e.g., ≈5 mV for the extracellular K+ level and ≈150 mV for the intracellular K+ level).^1^ The polarization of the potassium transmembrane potential is involved in diverse biological processes, such as cell growth, metabolism, and apoptosis.^2^ For this reason, the development of potassium homeostasis disturbers is a novel approach to cause dysfunction in cells. With regard to synthetic potassium ionophores, 18‐crown‐6 ether has been exploited because of the pore size and because electron‐donating oxygen atoms possess a strong affinity with potassium.^3^ Crown ether‐bearing ionophores act as an artificial potassium ion transporter and selectively transport potassium ions, which can broaden their biomedical applications.[qv: 3d,4] In terms of a drug, potassium ionophores impart an apoptosis‐inducing ability by overproducing reactive oxygen species (ROS) because perturbed potassium homeostasis suppresses basic biological functions. Although synthetic potassium ionophores have been shown to have potential therapeutic applications as a novel apoptosis inducer, the apoptotic mechanism by perturbed potassium homeostasis is still unclear.^4^ Furthermore, the synthetic potassium ionophores possesses excessive high lipophilicity for cell‐binding affinity, which caused unsatisfactory applicability.^4^

**Figure 1:**
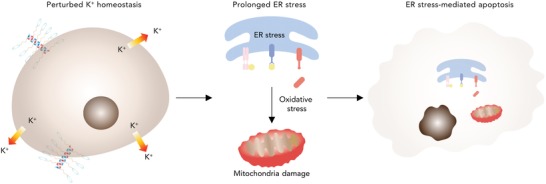
AIPs induce ER‐mediated apoptosis by disturbing potassium homoeostasis. Proposed mechanism for ER stress‐mediated apoptosis driven by the perturbation of potassium homeostasis. AIPs reduce the intracellular potassium ion levels, resulting in strong activation of the unfolded protein response (UPR) in the ER. The stressed ER leads to oxidative conditions by overproducing ROS, thereby damaging the mitochondria and activating apoptosis signaling.

Herein, we developed endoplasmic reticulum (ER) stress‐mediated apoptosis‐inducing polypeptides (AIPs) driven by perturbing potassium homeostasis and demonstrated the detailed mechanism of ER stress‐mediated apoptosis using AIPs (**Figure** **1**). We synthesized synthetic helical polypeptides by elongating the side chains, and conjugated the functional moieties (18‐crown‐6, trimethyl ammonium and hydrocarbon chain). Furthermore, we differentiated the hydrophobic chain length to find the optimal AIPs that can effectively bind to cell membranes. Our hypothesis was that the hydrophobic domain of AIPs was capable of contributing to binding affinity to cell membranes. The cationic helical structure enabled AIPs to interact with cell membranes. Moreover, the 18‐crown‐6 moiety was capable of transporting potassium ions. Due to the structural characteristics, the AIPs disturbed potassium homeostasis while intracellular calcium levels were elevated, resulting in the severe disturbance of ion homeostasis (Figure 1). The perturbed potassium homeostasis strongly inflicted prolonged ER stress on the cells, thereby initiating the onset of programmed cell death (Figure 1). The stressed ER fostered the amplified oxidative conditions and inflicted severe damage on the mitochondria, resulting in activation of apoptosis (Figure 1). In this study, we demonstrated ER stress‐mediated apoptosis pathways by perturbing potassium homeostasis in vitro and in vivo. To our knowledge, this study provides the first evidence showing that perturbed potassium homeostasis results in ER stress‐mediated apoptosis.

## 2 Results

### 2.1 Characterization of the AIPs

**Figure 2:**
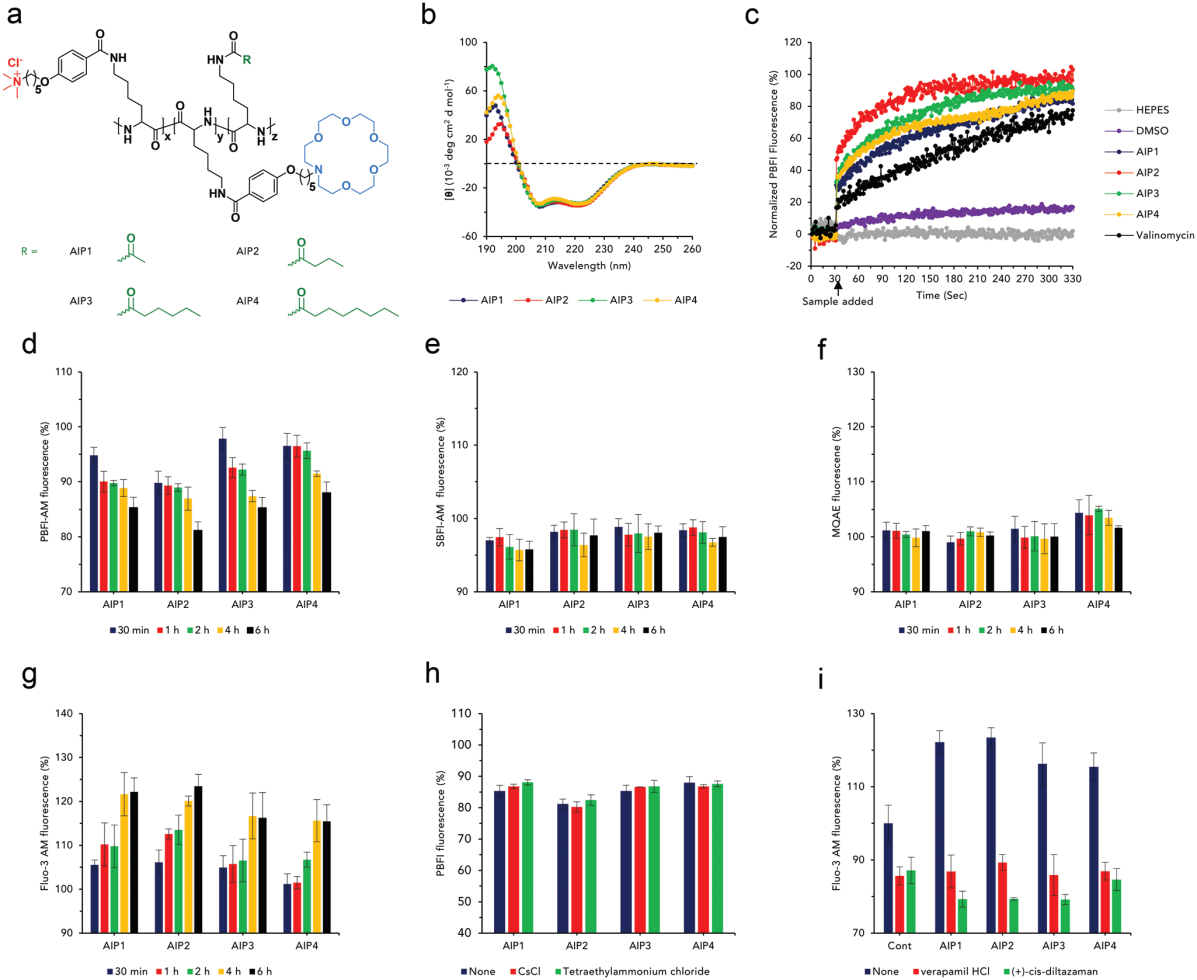
Characterization and ion transport activity of the AIPs. a) Chemical structure of the AIPs, differentiated by the hydrocarbon chain length. b) CD spectra of the AIPs. c) Potassium ion transport kinetics of the AIPs, valinomycin, DMSO, and HEPES using PBFI‐loaded LUVs. Time‐dependent relative intracellular ion measurements after treatment with AIPs (*n* = 3, S.D.). d) Potassium, e) sodium, f) chloride, and g) calcium. The cells were loaded with the corresponding ion‐sensitive fluorescence probe (PBFI‐AM, SBFI‐AM, MQAE, and Fluo‐3 AM). The relative potassium and calcium ion levels in the presence of potassium and calcium ion channel blockers (*n* = 3, S.D.). h) Potassium and i) calcium [CsCl, tetraethylammonium chloride; potassium channel blocker, verapamil HCl, (+)‐*cis*‐diltiazem HCl; calcium channel blockers].

Poly‐l‐lysine protected by two different protecting groups (TFA and Cbz) was synthesized by *N*‐carboxyanhydride (NCA) random copolymerization to selectively conjugate the building block to the primary amine groups (Figure S1, Supporting Information). Then, 1‐aza‐18‐crown‐6 ether and trimethylammonium moieties were attached to the peripheral elongated side chain after TFA deprotection to transport potassium and have a strong interaction with lipid plasma membranes (Figure S1, Supporting Information). In the last step, the hydrocarbon length was differentiated to find the optimal AIP after Cbz deprotection (**Figure** **2**a; Figure S1, Supporting Information). The detailed synthetic procedures and physical characterization are described in the Supporting Information (Figures S1–S13).

Circular dichroism (CD) spectrometry was employed to determine the protein secondary conformation of the AIPs (Figure 2b). The AIPs possessed intact helical conformations regardless of the hydrocarbon chain length (Figure 2b). Additionally, the secondary conformation of the AIPs was not influenced by potassium and large unilamellar vesicles (LUVs) (Figures S14 and S15, Supporting Information). The cytotoxic effect of the AIPs was evaluated to verify that the AIPs had a cytotoxic effect on cells and obtain the half maximal inhibitory concentration (IC_50_) value for each one (Figures S16–S18, Table S1, Supporting Information). Overall, the cell cytotoxicity was proportional to the AIP concentrations, and low IC_50_ values for the AIPs were obtained in NCI‐H460 cells compared with those of other cell lines (Table S1, Supporting Information). Therefore, NCI‐H460 cells were used for further studies.

### 2.2 Ion Transport Activity of the AIPs

We prepared 8‐hydroxypyrene‐1,3,6‐trisulfonic acid trisodium salt (HPTS)‐loaded LUVs to demonstrate the potassium ion transport activity (Figure S19, Supporting Information). All the normalized fluorescence intensities, *I*_F_, were dramatically increased, and the time‐ and concentration‐dependent increase in *I*_F_ indicated that the AIPs possessed ion transport activity (Figure S19, Supporting Information). Moreover, the AIPs had a high cation selectivity for potassium ions, while no anion selectivity was observed (Figures S20 and S21, Supporting Information). Using potassium‐binding benzofuran isophthalate (PBFI)‐loaded LUVs, we directly evaluated the potassium ion transport activity to verify the potassium influx kinetics of the AIPs (Figure 2c). The PBFI fluorescence intensity of the AIPs was rapidly elevated immediately after AIP treatment (Figure 2c). Although valinomycin also triggered a potassium influx into the LUVs, the influx kinetics were somewhat retarded compared to those of the AIPs (Figure 2c). Based on the result, AIPs rapidly transported potassium cations with the high selectivity to potassium. A single channel analysis was performed to verify the ion transport mechanism of the AIPs (Figure S22, Supporting Information). The change in current was almost negligible in the AIPs, indicating that the AIPs had a strong tendency to transport potassium without forming ion channels (Figure S22, Supporting Information). Consequently, the AIPs would show behavior similar to ion carriers rather than ion channels when transporting potassium ions.

To confirm the reduction of the intracellular potassium levels in the cells, we quantified the intracellular potassium levels in a time‐dependent manner using a potassium fluorescence indicator (PBFI‐AM; PBFI acetoxymethyl ester) (Figure 2d; Figure S23a, Supporting Information). The intracellular potassium levels of the AIP groups were gradually reduced (Figure 2d). Among the AIPs, AIP2 possessed a strong capability to reduce the potassium ion level. Furthermore, prevalent ions (sodium and chloride) were measured using fluorescence indicators to determine if AIPs affect the homeostasis of other ions [SBFI‐AM; sodium‐binding benzofuran isophthalate acetoxymethyl ester, MQAE; *N*‐(ethoxycarbonylmethyl)‐6‐methoxyquinolinium bromide] (Figure 2e,f; Figure S23b,c, Supporting Information). At intracellular sodium levels, the relative SBFI‐AM fluorescence intensity of the AIPs was maintained almost consistently in a time‐independent manner (Figure 2e). Likewise, the MQAE levels of the AIPs were maintained (Figure 2f). To verify the calcium influx induced by the perturbed potassium homeostasis, we assessed the intracellular calcium concentrations using Fluo‐3 AM in a time course assay (Figure 2g; Figure S23d, Supporting Information). The Fluo‐3 AM fluorescence levels of the AIPs were gradually augmented, indicating that the disturbance of potassium homeostasis induced the calcium influx into the cytosol (Figure 2g).

Intracellular potassium concentrations were evaluated under the inhibition of the potassium channels to demonstrate that the AIPs perturbed the potassium homeostasis by themselves without stimulating the potassium channels (Figure 2h). Cesium chloride and tetraethylammonium chloride, potassium channel blockers, were added along with the AIPs. The relative PBFI‐AM fluorescence levels of the AIPs were almost analogous, indicating that the AIPs spontaneously drove out the potassium ions (Figure 2h). Moreover, the intracellular calcium levels of the AIPs under calcium channel‐blocking conditions were determined and revealed strong activation of calcium signaling (Figure 2i). The intracellular calcium concentrations of the AIPs in the presence of a calcium channel blocker [verapamil HCl and (+)‐*cis*‐diltiazem HCl] were dramatically reduced compared to those without a calcium channel blocker, indicating that the perturbed potassium homeostasis activated calcium channels that transported calcium ions into the cytosol (Figure 2i).

### 2.3 AIP Trafficking

**Figure 3:**
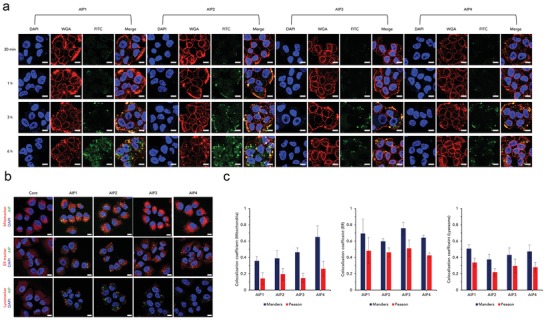
Intracellular trafficking of the AIPs visualized by CLSM. a) Visualization of the AIPs binding to lipid plasma membranes at 0.5, 1, 3, and 6 h (red; WGA, green; AIPs, blue; DAPI, scale bar; 10 µm). b) AIPs targeting intracellular organelles, mitochondria, ER, and lysosomes (scale bar; 10 µm). c) Colocalization coefficient of the AIPs in the mitochondria, ER and lysosomes, analyzed by ZEISS CLSM software (*n* = 3, S.D.). Colocalization coefficient values were determined by the methods of Manders and Pearson.

Confocal laser scanning microscopy (CLSM) was used to demonstrate the internalization mechanism (**Figure** **3**a). The lipid plasma membranes were stained with Alexa Fluor 555 wheat germ agglutinin (WGA) to confirm the colocalization of the FITC‐labeled AIPs (Figure 3a). As shown in Figure 3a, the green fluorescence of the AIPs was increasingly overlapped with the red fluorescence until 3 h, indicating that the AIP molecules electrostatically interacted with the lipid plasma membranes. After 6 h, green fluorescence signals were frequently observed in the cytosol rather than in the lipid membranes (Figure 3a). Among the AIPs, AIP1 and AIP2 highly bound to the lipid plasma membranes at the early stage (≈3 h). However, intense green fluorescence signals were observed in the cytosol at 6 h (Figure 3a). Additionally, flow cytometry was employed to confirm the AIPs binding affinity with cell membranes. Among AIPs, AIP2 highly interacted with the lipid plasma membranes, implying that the proper hydrophobic domain remarkably imparted the anchoring capability (Figure S24, Supporting Information).

To evaluate the targetability to intracellular organelles, we visualized the cells by staining the mitochondria, ER and lysosomes (Figure 3b). As shown in Figure 3b, the internalized AIPs were not colocalized within the mitochondria, ER, and lysosomes because the green fluorescence was not merged with the red fluorescence. To confirm the targeting capability, we calculated each colocalization coefficient by the methods of Manders and Pearson (Figure 3c). The positive range of the Manders coefficient was 0.5–1, while that of the Pearson coefficient was over 0.5.^5^ Given this factor, the AIPs did not target the mitochondria and lysosomes because both coefficient values were in the negative range (Figure 3c). In the case of the ER, based on the Pearson coefficient values of the AIPs, they were not localized in the ER, although the Manders coefficient values of the AIPs were slightly over 0.5 (Figure 3c). Consequently, we confirmed that the AIPs did not have an organelle‐targeting ability and remained in the cytoplasm.

### 2.4 ER Stress by Perturbing Potassium Homeostasis

**Figure 4:**
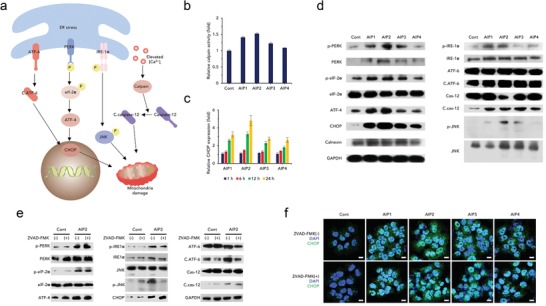
Perturbed potassium homeostasis imposes ER stress regardless of the caspase pathways. a) Proposed mechanism of the ER stress signaling pathway inducing apoptosis. b) Relative calpain activity assay after treatment with the AIPs (0.25 × 10^−6^ m) (*n* = 3, S.D.). c) ER stress signal pathways shown by Western blotting of ER stress‐related proteins. GAPDH was used as a loading control. d) Relative expression levels of CHOP measured at 0.5, 1, 6, and 12 h using CLSM (*n* = 3, S.D.). e) Verification of ER stress signals not regulated by caspase‐dependent pathways. Western blotting of control and AIP2 groups, performed in the presence or absence of ZVAD‐FMK (40 × 10^−6^ m). f) Immunofluorescence images of CHOP in the presence or absence of ZVAD‐FMK (40 × 10^−6^ m) (scale bar; 10 µm). Nuclei were stained with DAPI (blue fluorescence). CHOP was stained with Alexa Fluor 488 (green fluorescence). **p < 0.01, ***p < 0.005, ****p < 0.0001 (compared to control) (*t*‐test).

We postulated that AIP disturbing ion homeostasis resulted in severe ER stress, thereby inducing apoptosis (**Figure** **4**a). First, a calpain activity assay was performed to verify the increase in the intracellular calcium level. The calpain in all the AIP groups was slightly expressed compared to that in the untreated group because of the elevation of the intracellular calcium levels (Figure 4b). As shown in Figure 4c, cleaved caspase‐12 was detected after treatment with the AIPs, suggesting that the activated calpain resulted in the activation of caspase‐12. To verify ER stress, we performed Western blotting. Treatment with the AIPs resulted in the phosphorylation of inositol‐requiring enzyme 1α (IRE‐1α) and protein kinase RNA‐like ER kinase (PERK) and the dissociation of activating transcription factor 6 (ATF‐6), initiating ER stress (Figure 4c). Furthermore, eukaryotic initiation factor 2α (eIf‐2α) was phosphorylated, and then, ATF‐4 was highly expressed in the AIP‐treated groups, indicating the activation of PERK signaling^6^ (Figure 4c). In the case of IRE1α signaling, phosphorylated IRE1α (p‐IRE‐1α) provokes the phosphorylation of c‐Jun N‐terminal kinases (JNK)[qv: 6b] (Figure 4c). A band for phosphorylated JNK (p‐JNK) was observed in the AIP‐treated groups, indicating that the AIPs also affected IRE1α signaling (Figure 4c). Based on these results, AIP2 strongly acted as an ER stress inducer due to the considerable activation of both the PERK and IRE1α signaling pathways.

At the end stage of ER stress, ATF‐4 and cleaved ATF‐6 enable C/‐EBP homologous protein (CHOP) to be highly expressed.^7^ AIPs increased the expression level of CHOP (Figure 4c). Moreover, the CHOP expression level was gradually increased in a time‐dependent manner (Figure 4d; Figure S25, Supporting Information). AIP2 strongly triggered CHOP production due to the substantial induction of ER stress. Therefore, AIP2 possessed an outstanding capability to provoke ER stress and was exploited as the optimal ER stress inducer for further study.

To confirm whether ER stress was controlled by caspases, we detected ER‐mediated proteins under caspase‐inhibiting conditions (Figure 4e). Overall, ER stress pathways were independent on the activation of caspases except for activation of ATF‐6 and JNK. (Figure 4e). Additionally, the degree of CHOP expression was detected and visualized by CLSM in the presence or absence of ZVAD‐FMK (Figure 4f). An undetectable difference between conditions “with” and “without ZVAD‐FMK” was observed in all the AIP groups (Figure 4f). Based on these results, AIP2 strongly induced ER stress in the cells regardless of the status of the caspase pathways.

### 2.5 Proapoptotic Activity Driven by ER Stress

**Figure 5:**
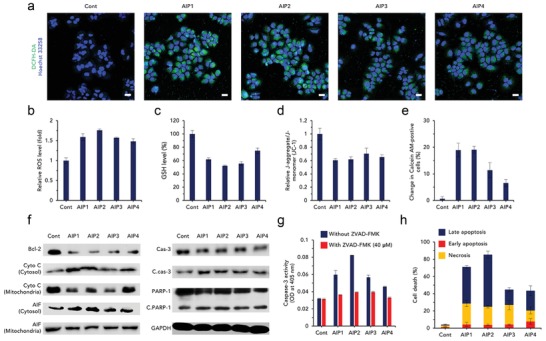
Induction of ER stress‐mediated apoptosis by oxidative stress. a) Visualization of ROS using DCFH‐DA after treatment with AIPs (0.25 × 10^−6^ m) (scale bar; 20 µm). Quantification of b) ROS (fold; comparison to untreated group) and c) GSH level (%) to verify the presence of oxidative environments (*n* = 3, S.D.). Disruption of mitochondria by oxidative stress. d) JC‐1 assay for depolarization of mitochondrial membrane potential (*n* = 3, S.D.). e) Mitochondria transition pore assay expressed as the change in calcein‐AM‐positive cells (*n* = 3, S.D.). f) Immunoblotting of apoptosis‐related proteins (Bcl‐2, Cyto C: cytochrome C, C.cas‐3: cleaved caspase‐3, Cas‐3: caspase‐3, C.PARP‐I: cleaved PARP‐I). Glyceraldehyde 3‐phosphate dehydrogenase (GAPDH) was used as a protein loading control. g) Caspase‐3 activity using Ac‐DEVD‐p‐nitroaniline, a caspase‐3 substrate, quantified by optical density (OD) at 405 nm (*n* = 3, S.D.). h) Cell death evaluated by flow cytometry after FITC‐annexin V and PI staining (*n* = 4, S.D.). **p < 0.01, ***p < 0.005, ****p < 0.0001 (compared to control) (*t*‐test).

We hypothesized that AIPs induced ER stress‐mediated apoptosis by disturbing ion homeostasis because severe ER stress initiates the onset of apoptosis.^7^ To prove our hypothesis, we first measured intracellular ROS levels of the AIP groups to demonstrate the acceleration of oxidative conditions (**Figure** **5**a,b). The ROS levels of the AIPs were substantially augmented compared to those of the untreated group, indicating that the oxidative environments became increasingly intensified (Figure 5b). Similarly, in the CLSM images, the green fluorescence intensity of the AIP‐treated groups was much more intense than that of the untreated group (Figure 5a). Additionally, the glutathione (GSH) levels of the AIP groups were analyzed to confirm the decrease in the intracellular GSH concentrations (Figure 5c). As expected, the intracellular GSH concentrations of the AIPs were highly reduced by excessive ROS production (Figure 5c).

Mitochondria‐related assays were conducted to verify the dysfunction of the mitochondria. An assay for depolarization of the mitochondrial membrane potential was performed to confirm the destabilization of the mitochondrial membranes (Figure 5d). The relative J‐monomer/J‐aggregate ratios of the AIPs were relatively detrimental compared with those of the untreated group, indicating that oxidative stress triggered severe mitochondrial dysfunction (Figure 5d). To confirm the loss of mitochondrial function, we conducted a mitochondrial transition pore assay (Figure 5e). The change in calcein‐AM, which is directly proportional to the degree of mitochondria permeability, was remarkably increased in the AIP‐treated groups (Figure 5e). Among the AIPs, AIP2 severely damaged the mitochondria by activating the oxidative environments (Figure 5e). Moreover, oxidative stress had a detrimental effect on nuclei, resulting in nuclear fragmentation, as visualized by CLSM (Figure S26, Supporting Information).

Damaged mitochondria activate caspase‐dependent apoptotic signaling.^8^ To demonstrate the caspase‐dependent apoptotic activity, we evaluated caspase‐3 activity using a caspase‐3 substrate (Figure 5g). The AIPs cleaved the caspase‐3 compared with the control, suggesting that caspase‐dependent apoptosis did occur (Figure 5g). Among the AIPs, AIP2 strongly induced caspase‐3 due to severe oxidative stress (Figure 5g). However, in the presence of a caspase inhibitor, ZVAD‐FMK, the caspase‐3 activity of the AIPs was attenuated (Figure 5g).

The mitochondria‐dependent apoptotic pathway was revealed by Western blotting (Figure 5f). As shown in Figure 5f, the expression of B cell lymphoma‐2 (Bcl‐2) was highly inhibited, suggesting that mitochondria‐dependent apoptosis was initiated. The suppression of Bcl‐2 caused cytochrome C to be released from the mitochondria. The AIP‐treated groups released cytochrome C from the mitochondria because the cytosolic cytochrome C was highly expressed, while the expression of mitochondrial cytochrome C was reduced (Figure 5f). In general, elevated cytosolic cytochrome C consecutively cleaves caspase‐3 and poly(ADP‐ribose) polymerase‐1 (PARP‐1).^8^ The cleaved forms of caspase‐3 and PARP‐1 are shown in Figure 5f, indicating that apoptosis was significantly triggered. Additionally, the expression of apoptosis‐inducing factor (AIF) released from the mitochondria was evaluated to confirm whether caspase‐independent pathways were involved (Figure 5f, Figure S27, Supporting Information). The AIPs also induced caspase‐independent apoptosis, as shown by the release of AIF (Figure 5f). Based on the immunoblot assay, the AIPs highly induced caspase‐dependent and caspase‐independent apoptosis.

Oxidative stress could strongly activate proapoptotic signaling, resulting in programmed cell death. To evaluate the degree of apoptotic events, we performed an apoptosis assay using fluorescein isothiocyanate (FITC)‐annexin V and propidium iodide (PI) staining (Figure 5h; Figure S28, Supporting Information). The AIP‐treated cells accounted for a considerable proportion of the apoptotic cells, while the number of necrotic cells was extremely low, implying that programmed cell death was elicited (Figure 5h). In the AIP‐treated groups, late apoptosis was stronger in the AIP2 group than the other AIP‐treated groups (Figure 5h).

### 2.6 Confirmation of ER Stress‐Mediated Apoptosis Using a Tumor‐Bearing Mouse Model

**Figure 6:**
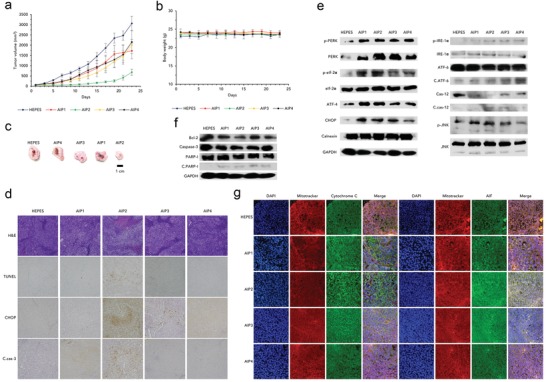
Induction of ER stress‐mediated apoptosis in vivo using a tumor‐bearing mouse model. Changes in a) tumor volume and b) body weight after treatment with HEPES and AIPs (2 mg kg^−1^). (AIP1, AIP2 and AIP3: *n* = 6, AIP4: *n* = 4, tumor volume; S.E., body weight; S.D.) c) Optical images of the excised tumor tissues after all mice were sacrificed on day 23. d) Immunohistochemical assays of the harvested tumor sections for H&E, TUNEL, CHOP, and c.cas‐3: cleaved caspase‐3. All images were taken by an optical microscope (magnification: 400 ×). Immunoblot assays of the harvested tumor tissues for e) ER stress‐related and f) apoptosis‐related proteins. GAPDH was used as a loading control. g) Immunofluorescence of cyto C (green fluorescence) and AIF (green fluorescence) using the excised tumor tissues stained with DAPI (blue fluorescence) and Mitotracker Red (red fluorescence). All images were obtained by CLSM (magnification: 400×). *p < 0.05, ****p < 0.0001 (compared to control) (*t*‐test).

To confirm that AIPs induced ER stress‐mediated apoptosis in vivo, we subcutaneously implanted NCI‐H460 cells into nude BALB/c male mice. The AIP‐treated groups showed retarded tumor growth compared to the HEPES group, while the body weights of all the groups were consistently maintained, indicating that the AIPs imparted a cytotoxic effect only on the tumors (**Figure** **6**a,b). Among the AIPs, AIP2 possessed the highest tumor‐inhibiting capability because tumor proliferation in the mouse model treated with AIP2 was almost suppressed (Figure 6a). For the excised tumor tissues, the tumor volume of the AIP2‐treated group was substantially reduced compared to that of the other groups (Figure 6c). Moreover, AIP2 strongly damaged tumor tissues compared with those of the other groups, as shown by the hematoxylin and eosin (H&E) images (Figure 6d).

To verify the suppression of tumor proliferation by ER stress‐mediated apoptosis, we performed several assays (Western blotting and immunohistochemistry) (Figure 6d,e). Similar to the in vitro experiments, the AIPs induced ER stress in the tumors because the ER stress sensors were activated (Figure 6e). In addition, caspase‐12 was cleaved, indicating that the intracellular calcium level was increased (Figure 6e). The activated PERK pathway phosphorylated eIf‐2α, leading to the high consecutive expression of ATF‐4 and CHOP. Additionally, the phosphorylation of IRE‐1α resulted in the phosphorylation of JNK (Figure 6e). Among the AIPs, AIP2 strongly induced ER stress in the tumors because ER stress‐related proteins were strongly produced or phosphorylated (Figure 6e). Even in the immunohistochemistry images of CHOP, AIP2 stimulated CHOP expression (Figure 6d). Consequently, the AIPs strongly induced ER stress in the tumor, resulting in the initiation of apoptosis.

To verify ER stress‐mediated apoptosis, we detected apoptosis‐related proteins (Figure 6f,g). As shown in Figure 6f, Bcl‐2 expression was inhibited in the AIP‐treated groups, which led to mitochondria‐dependent apoptosis. The damaged mitochondria released AIF and cytochrome C, which was confirmed by the immunofluorescence images (Figure 6g). The overlapping proportion of green and red fluorescence was remarkably diminished compared to that of the HEPES group, and the green fluorescence of the AIP‐treated groups became more intense, which could indicate the release of cytochrome C and AIF from the mitochondria (Figure 6g). The released cytochrome C led to the consecutive cleavage of caspase‐3 and PARP‐1, which accelerated apoptosis (Figure 6d,f). AIP2 strongly induced ER stress‐mediated apoptosis compared to the other AIPs because of its more active apoptosis cascade (Figure 6d,f). Finally, a TUNEL assay was performed to confirm the apoptotic events of the AIPs. Apoptosis was induced in the AIP1, AIP2, and AIP3‐treated groups (the effects of AIP4 were negligible) (Figure 6d). Among the AIPs, AIP2 induced the highest proportion of apoptotic events (Figure 6d).

To verify the in vivo applicability of AIPs as a potential drug, we performed several important assays (biodistribution, toxicity assays) (Figures S29–S31, Supporting Information). First, near‐infrared fluorescence (NIRF) imaging was employed to verify AIP trafficking in vivo (Figure S29, Supporting Information). All AIP molecules accumulated in all organs after 48 h (Figure S29c, Supporting Information). We found that AIP2 molecules were relatively localized within tumor tissues compared to those of the other groups (Figure S29a, Supporting Information). Although the NIRF signal was detected in the organs, severe cytotoxic effects were not observed in H&E images of all the harvested organs (Figure S30, Supporting Information). Compared to the HEPES groups, all the AIP groups did not show damage in the major organs, as demonstrated by the inappreciable difference between the HEPES and AIP‐treated groups, excluding the liver of the AIP1 group; liver toxicity is shown (Figure S30, Supporting Information).

Moreover, aspartate aminotransferase (AST), alanine aminotransferase (ALT), creatinine, and blood urea nitrogen (BUN) levels were measured in the collected blood to assess the toxicity against the kidney and liver (Figure S31, Supporting Information). For all the groups, the levels of ALT and AST (for liver toxicity) were within the normal range (Figure S31, Supporting Information). Likewise, those of BUN and creatinine (for kidney toxicity) were in the normal range (Figure S31, Supporting Information). In addition, a hemolysis assay was conducted to assess the blood compatibility (Figure S32, Supporting Information). All the AIPs did not lyse red blood cells because hemolytic activity was rare (Figure S32, Supporting Information). Based on these results, we concluded that AIPs possessed outstanding in vivo applicability owing to their superior biocompatibility. To demonstrate that AIPs were delivered to tumor tissues via their vascularity, the immunohistological assay was performed by CD31 antibody staining. As shown in Figure S33 (Supporting Information), compared to HEPES group, the proportion of blood vessels around tumor was highly reduced, meaning that AIPs deactivated angiogenesis. Although AIPs were translocated to tumors via the blood vessels, AIPs suppressed the tumor vascularity by ER stress‐mediated apoptosis (Figure S33, Supporting Information). Among AIPs, AIP2 remarkably inhibited the expression of CD31 (Figure S33, Supporting Information). Finally, the immunoblotting of angiogenesis‐related proteins (VEGF, vascular endothelial growth factor; MMP‐2, matrix metalloproteinase‐2; MMP‐9, matrix metalloproteinase‐9) were carried out to demonstrate the inhibition of angiogenesis by treating AIPs (Figure S34, Supporting Information). Compared to control, AIPs‐treated groups exhibited that the expression of angiogenesis‐related proteins were down‐regulated, meaning that AIPs inhibited angiogenesis (Figure S34, Supporting Information).

## 3 Conclusion

The disturbance of ion homeostasis is a novel strategy for the acceleration of cell malfunctions.^9^ The disturbed ion homeostasis typically elicits apoptotic activity by ion transportation.^9^ Considering the general apoptotic mechanism, ROS are overproduced, inducing oxidative stress in cells.^9^ The severe oxidative conditions inflict damage to mitochondria and then activate mitochondria‐dependent apoptosis pathways.^9^ Although ionophore‐based apoptosis inducers have been recently investigated, the apoptotic mechanisms are not fully understood. Moreover, the induction of apoptosis by synthetic ionophores was not verified in vivo due to the lack of bioapplicability. Therefore, the mechanism of apoptosis induced by disturbing ion homeostasis should be elucidated, and the practicability of ionophores should be determined.

In this study, we introduced a crown ether moiety in the polypeptide to impart water solubility and potassium transport characteristics. In addition, the tetramethylammonium group and hydrocarbon chain allowed for interactions between lipid plasma membranes and the polypeptide. Because of their structural features, AIPs possess potassium ion transport activity. When AIPs were tested in a cellular environment, we hypothesized that AIPs perturbed potassium homeostasis, inducing ER stress‐mediated apoptosis. As expected, AIPs perturbed potassium homeostasis and accelerated calcium influx into the cytosol. ER stress‐related proteins were detected to confirm the stimulation of ER stress by disturbed potassium homeostasis. The results indicate that perturbed ion homeostasis induces the unfolded protein responses in the ER and then highly stimulates the ER stress‐related signaling cascade for apoptosis. Furthermore, ER stress‐related proteins were detected under caspase‐blocking conditions to confirm whether ER stress pathways were governed by the caspase cascade. Overall, ER pathways were not affected by caspase signaling, indicating that ER stress is generated prior to apoptosis activation. We assessed the pathways of ER stress‐mediated apoptosis by immunoblot and apoptosis‐related assays. Stressed ER exerted oxidative stress in the mitochondria and then stimulated mitochondria‐dependent apoptosis. Finally, we evaluated the apoptotic activity in vivo to demonstrate its practicability as a therapeutic. All the in vivo results imply that AIP2 strongly inhibited tumor growth by ER stress‐mediated apoptosis without any side effects and might be used as a potential anticancer agent.

In conclusion, we developed a new potassium ionophore that induces ER stress‐mediated apoptosis and revealed detailed apoptotic mechanisms. This study presented the first evidence that the perturbation of potassium homeostasis strongly activated ER stress–induced apoptosis. Moreover, the strategy for disturbing potassium homeostasis will be harnessed for future anticancer drugs.

## 4 Experimental Section

*Determination of the AIP Secondary Structures*: A CD spectrometer (J‐815 spectropolarimeter 150‐L type, JASCO, Japan) was used to characterize the secondary protein structure using a quartz cell with a 0.02 mm path length in the range of 200–260 nm at RT. The CD spectra were recorded with 100 nm min^−1^ scanning, 1 nm bandwidth, 4 s response time, 1.0 nm data pitch, and 10 accumulations. All the AIP samples were dissolved in deionized water, and all the concentrations were adjusted to 1 mg mL^−1^.

*Potassium Ion Transport Activity*: A phospholipid thin film (3 mL in 20 mg mL^−1^ EYPC chloroform solution) was prepared by the procedure described above.[qv: 9d] For the potassium influx, the LUV solution (2 mL in 10 × 10^−3^ m HEPES, 100 × 10^−3^ m Et_4_NCl and 75 × 10^−6^ m PBFI) was dialyzed against a HEPES buffer solution (10 × 10^−3^ m HEPES and 100 × 10^−3^ m KCl). The AIP series (0.25 × 10^−6^ m), DMSO (10 µL), and valinomycin (0.25 × 10^−6^ m) were added to the LUV solution at *t* = 30 s. Time‐dependent fluorescence intensity was measured by a fluorescence spectrometer for 330 s (λ_ex_ = 340 nm, λ_em_ = 500 nm and time interval: 1 s; Gemini XPS microplate reader, MOLECULAR DEVICES, USA). PBFI influx (%) was calculated as follows: (*F*_t_ − *F*_0_)/(*F*_s_ − *F*_0_) × 100 (%).

*Measurement of Intracellular Ion Concentrations*: NCI‐H460 cells (10 000 cells per well in 96‐well plate) were pre‐stained with PBFI‐AM, SBFI‐AM (9 × 10^−6^ m in 0.04% Pluronic F127‐containing PBS, ThermoFisher Scientific, USA), Fluo‐3 AM (0.9 × 10^−6^ m in DMSO, Thermo Scientific, USA), or MQAE (20 µL, 9 × 10^−6^ m in 0.04% Pluronic F127‐containing PBS, Santa Cruz Biotech, USA) for 1.5 h (PBFI‐AM, SBFI‐AM) or 30 min. (Fluo‐3 AM, MQAE) and then washed with PBS three times. DMEM was added to each well before the treatment with the AIP series (0.25 × 10^−6^ m). Each fluorescence intensity was measured at the predetermined time points by a fluorescence spectrometer (PBFI‐AM, SBFI‐AM; λ_ex_ = 340 nm, λ_em_ = 500 nm, Fluo‐3 AM; λ_ex_ = 506 nm, λ_em_ = 526 nm, MQAE; λ_ex_ = 350 nm, λ_em_ = 460 nm) (Gemini XPS microplate reader, MOLECULAR DEVICES, USA). For the detection of intracellular potassium levels under inhibiting conditions, NCI‐H460 cells were cotreated with the AIP series (0.25 × 10^−6^ m), tetraethylammonium chloride (1 × 10^−3^ m, Tokyo Chemical Industry Co., LTD., Japan) and cesium chloride (1 × 10^−3^ m, Daejung Chemical, Korea) after PBFI‐AM staining. The intracellular potassium level (%) was expressed as *F*_sample_/*F*_cont_ × 100 (%). For the detection of the intracellular calcium levels under inhibiting conditions, NCI‐H460 cells were co‐treated with the AIP series (0.25 × 10^−6^ m) and calcium channel blockers ((+)‐*cis*‐diltiazem HCl (1 × 10^−6^ m, Tokyo Chemical Industry Co., LTD., Japan), or verapamil HCl (1 × 10^−6^ m, Tokyo Chemical Industry Co., LTD., Japan)) after Fluoro‐3 AM staining. The intracellular calcium level (%) was expressed as *F*_sample_/*F*_cont_ × 100 (%).

*CLSM Observation of Plasma Membranes*: NCI‐H460 cells (80 000 cells per well in a 24‐well plate) were seeded onto a cover slip for a 24‐well plate and then stabilized for 1 d. FITC‐labelled AIP series (25 × 10^−9^ m) were added to each well after washing with PBS three times and then further incubated for 0.5, 1, 3, and 6 h. Each well was carefully rinsed with PBS three times. In the following step, the cells were fixed by a 4% paraformaldehyde solution for 10 min. Alexa Fluor 555 conjugate of the WGA staining solution (5 µg mL^−1^ in PBS, ThermoFisher Scientific, USA) was added to each well for 5 min., and then, the cells were washed with PBS three times prior to DAPI staining (300 × 10^−9^ m). The cell images were obtained by CLSM (LSM 800 META, ZEISS, Germany).

*CLSM Observation of the Organelles*: NCI‐H460 cells (80 000 cells per well in a 24‐well plate) were seeded onto a cover slip for a 24‐well plate and then stabilized for 1 d. For the lysosome, mitochondria, and ER visualization, the cells were pre‐stained with Lysotracker deep red (50 × 10^−9^ m, ThermoFisher Scientific, USA), Mitotracker deep red (450 × 10^−9^ m, ThermoFisher Scientific, USA) or ER tracker Red (100 × 10^−9^ m, ThermoFisher Scientific, USA). The stained cells were treated with FITC‐labelled AIPs (25 × 10^−9^ m) for 3 h before extracellular green fluorescence was quenched by trypan blue (0.1 wt% in PBS). For mitochondria visualization, the cells were fixed by a 4% paraformaldehyde solution prior to DAPI staining (300 × 10^−9^ m). For lysosome and ER trafficking, the cells were treated with 4% FBS‐including DMEM after Hoechst 33 258 staining (5 µg mL^−1^ in PBS, ThermoFisher Scientific, USA). The cells were visualized by CLSM (LSM 800 META, ZEISS, Germany). The values of the Pearson and Manders colocalization coefficient were quantified by the ZEISS microscope software ZEN2. The error bars were expressed as S.D. based on three different images.

*Calpain Activity Assay*: NCI‐H460 cells (500 000 cells per well in 6‐well plates) were treated with the AIP series (0.25 × 10^−6^ m) for 24 h. The cells were lysed by a cold RIPA buffer, and then, the cell lysates were centrifuged to remove the cell debris. The protein concentration was adjusted to 1 mg mL^−1^ determined by a BCA kit using BSA as a standard. The calpain activity assay was determined with a commercial kit (Bio‐Vision, USA). The extracted proteins (50 µg) were mixed with a calpain substrate (Ac‐LLY‐AFC) and then incubated for 1 h at 37 ˚C in dark (total volume adjusted to 100 µL). The calpain activity was measured by a fluorescence spectrophotometer (λ_ex_ = 400 nm; λ_em_ = 505 nm) (Gemini XPS microplate reader, MOLECULAR DEVICES, USA).

*Western Blotting*: NCI‐H460 cells (500 000 cells per well in a 6‐well plate) were treated with the AIP series (0.25 × 10^−6^ m) for 1 d. Proteins were extracted using a cold RIPA buffer with 0.1% protease inhibitor (Sigma‐Aldrich, USA) and phosphatase inhibitor cocktails (Sigma‐Aldrich, USA). Electrophoresis was conducted by loading 50 µg of proteins into each well of a sodium dodecyl sulfate‐polyacrylamide gel (SDS‐PAGE) before the proteins were transferred to a PVDF membrane (0.2 µm pore size PVDF membrane, Roche, Swiss). The PVDF membranes were treated with the corresponding primary antibodies after blocking for 1 h (5 wt% BSA tris‐buffer saline with 0.05% Tween 20 (TBST) solution for phosphorylated proteins; 5 wt% skim milk TBST solution for general proteins). For apoptosis‐related proteins, c.caspase‐3 (1000:1, antirabbit polyclonal, Cell Signaling Technology, USA), caspase‐3 (1500:1, antirabbit polyclonal, Cell Signaling Technology, USA), PARP‐1 (6000:1, antirabbit polyclonal, Abcam, UK), Bcl‐2 (1000:1, antirabbit polyclonal, Santa Cruz Biotech, USA), and GAPDH (6000:1, antirabbit polyclonal, Santa Cruz Biotech, USA) were used. For ER stress‐related proteins, calnexin (6000:1, antirabbit polyclonal, Abcam, UK), IRE1α (3000:1, antirabbit polyclonal, Abcam, UK), p‐IRE1α (4000:1, antirabbit polyclonal, Abcam, UK), PERK (3000:1, antirabbit polyclonal, Abcam, UK), p‐PERK (3000:1, antirabbit monoclonal, Cell Science Technology, USA), ATF‐6 (3000:1, antirabbit polyclonal, Abcam, UK), caspase‐12 (2000:1, antirabbit monoclonal, Cell Science Technology, USA), ATF‐4 (2000:1, antirabbit polyclonal, Abcam, UK), p‐elF2α (4000:1, antirabbit polyclonal, Cell Signaling Technology, USA), elF2α (4000:1, antirabbit polyclonal, Cell Signaling Technology, UK), JNK (4000:1, antirabbit polyclonal, Cell Science Technology, USA), p‐JNK (4000:1, antirabbit polyclonal, Cell Science Technology, USA), CHOP (3000:1, antirabbit polyclonal, Abcam, UK) were used as primary antibodies. For general proteins, the primary antibody solutions were prepared in 5 wt% skim milk TBST solution. For phosphorylated proteins, the corresponding primary antibodies were diluted in 5 wt% BSA TBS‐T solution. The reactions were carried out overnight at 4 °C. HPR‐conjugated antirabbit used as a secondary antibody was added to the corresponding PVDF membranes before the overnight incubation at 4 °C. The blot signals were visualized by an enhanced chemiluminescent (ECL) reagent (GE healthcare, USA).

*Immunofluorescence Staining for ER Stress*: NCI‐H460 cells (80 000 cells per well in a 24‐well plate) seeded onto a coverslip were treated with the AIP series (0.25 × 10^−6^ m) for 1, 6, 12, and 24 h. For the CHOP activity in the presence of a caspase inhibitor, the cells were pre‐treated with ZVAD‐FMK (40 × 10^−6^ m) before the 1 d incubation with the AIPs (0.25 × 10^−6^ m). Thereafter, the cells were fixed by a 4% paraformaldehyde solution for 15 min. after washing with PBS three times. The cells were treated for 1 h with blocking solution (1% BSA, 0.3% triton‐X100 in phosphate buffer saline with 0.05% Tween 20 (PBST)) before being incubated with the corresponding primary antibody solution (1% bovine serum albumin (BSA), 0.3% Triton X‐100 in PBST and antirabbit CHOP; 1000:1) at 4 °C overnight. Alexa Fluor 488‐tagged secondary antibody solution (1% BSA, 0.3% Triton X‐100 in PBS and goat antirabbit; 1000:1) was treated for 2 h. In the last step, the cells were stained with DAPI (300 × 10^−9^ m in PBS) for 10 min. The immunofluorescence images were obtained by CLSM (LSM 800 META, ZEISS, Germany). For quantitative analysis, each arithmetic mean green fluorescence intensity of one cell in the nucleus was quantified by the ZEISS 2011 software (5 cells randomly selected). The time‐dependent relative expression of CHOP was calculated by the ratio of *F*_sample_ to *F*_cont_.

*Quantification of Intracellular ROS Levels*: NCI‐H460 cells were seeded on a 96‐well plate at 10 000 cells per well and incubated for 1 d to reach 90% confluency. The cells were treated with fresh DMEM containing the AIP solutions (0.25 and 0.75 × 10^−6^ m in PBS) for 24 h. ROS‐sensitive fluorescent dye and 2′,7′‐dichlorodihydrofluorescein diacetate (DCFH‐DA, 20 µL, 100 × 10^−6^ m in DMSO, Sigma‐Aldrich, USA) were added to each well and then incubated at 37 °C for 30 min. before the cells were rinsed with PBS three times. Intracellular ROS levels were measured by a fluorescence spectrometer (λ_ex_ = 495 nm, λ_em_ = 529 nm). The relative ROS level was expressed as the ratio of *F*_sample_ to *F*_control_.

*Visualization of the ROS Levels*: NCI‐H460 cells (100 000 cells per well) seeded onto coverslips for 24‐well plates were treated with the AIP series (61.25 × 10^−9^ m) for 12 h. For ROS imaging, DCFH‐DA (100 µL) was added to each well. The cells were washed with PBS three times to remove the excessive reagents before Hoechst 33 258 staining (5 µg mL^−1^ in PBS, ThermoFisher Scientific, USA). After nuclei staining, 10% FBS‐containing DMEM (500 µL) was added to the cells to prevent undesirable cell death. All the images were immediately taken by CLSM (LSM 800 META, ZEISS, Germany).

*Detection of the GSH Levels*: NCI‐H460 cells (150 000 cells per well in a 12‐well plate) were incubated for 1 d. The AIP series were added to each well (0.25 × 10^−6^ m in PBS) and then further incubated for 24 h before the cells were lysed with RIPA buffer at 4 °C. The cell lysates were centrifuged to isolate the supernatant. Ellman's reagent (20 µL, 0.75 × 10^−3^ m DTNB, Sigma‐Aldrich, USA) was added to the supernatant. The amount of GSH was quantified with a absorbance microplate reader at 405 nm. The GSH level was obtained by comparison with a nontreated group.^10^

*Depolarization of the Mitochondrial Membrane Potential*: NCI‐H460 cells were seeded onto a 96‐well plate at 10 000 cells per well and cultured for 1 d before the old medium was replaced with serum‐free medium containing the AIP series (0.25 and 0.75 × 10^−6^ m in PBS). After 24 h, the cells were treated with JC‐1 (5,5′,6,6′‐tetrachloro‐1,1′,3,3′‐tetraethylbenzimidazolycarbocyanine iodide) (10 µL, 100 µg mL^−1^ in DMSO, Invitrogen, USA) for 30 min. Then, the cells were rinsed with PBS three times. The fluorescence was detected by a fluorescence spectrometer (J‐monomer: λ_ex_ = 485 nm, λ_em_ = 530 nm; J‐aggregate: λ_ex_ = 535 nm, λ_em_ = 590 nm) (Gemini XPS microplate reader, MOLECULAR DEVICES, USA). The red/green fluorescence ratio (*R*) was calculated by the ratio of the fluorescence intensity (590/530 nm). The relative mitochondrial membrane depolarization was obtained by the ratio of *R*_sample_ to *R*_control_.

*Mitochondria Transition Pore Assay*: NCI‐H460 cells (150 000 cells per well in a 12‐well plate) were seeded and then stabilized for 1 d. The media were replaced with serum‐free medium containing the AIP series (0.25 × 10^−6^ m) and then further incubated for 24 h. Thereafter, the cells were isolated by consecutive trypsinization and centrifugation before they were diluted with HBSS/Ca (1 mL). Using a mitochondria transition pore assay kit (Thermo Scientific, USA), calcein AM (2 µL, 2 × 10^−6^ m in HBSS/Ca) and CoCl_2_ solution (2 µL, 80 × 10^−3^ m in saline) were added to all the cells. To abolish the cytosolic and mitochondrial calcein AM, ionomycin (2 µL, 100 × 10^−6^ m in DMSO) was additionally added to prepare the fully quenched cells. All the samples were incubated at 37 °C for 15 min. before the cells were washed with HBSS/Ca to remove the excessive staining and quenching reagents. The cells were diluted with DMEM (300 µL) for analysis by flow cytometry. The degree of mitochondrial transition pores was indirectly expressed as the difference between only cytosolic quenched cells (%) and fully quenched cells (%).

*Apoptosis Assay*: NCI‐H460 cells (150 000 cells per well in a 12‐well plate) were treated with the AIP series (0.25 × 10^−6^ m) for 1 d and then rinsed with PBS three times prior to trypsinization of the cells. Using an apoptosis assay kit (Thermo Scientific, USA), the cells were stained with propidium iodide (0.2 µg in 100 µg mL^−1^) and FITC‐annexin V (5 µL) dissolved in binding buffer (100 µL) and then incubated at RT for 10 min. The cells were diluted with DMEM (200 µL). Proapoptosis was evaluated by flow cytometry (Becton Dickinson and Company, Cytek FACSCalibur, USA).

*Isolation of the Cytosolic and Mitochondrial Fractions*: NCI‐H460 cells (500 000 cells per well in a 6‐well plate) were treated with the AIP series (0.25 × 10^−6^ m) for 1 d. The mitochondrial and cytosolic fractions were prepared following the manufacturer's instructions (Mitochondria Isolation Kit for Cultured Cells, ThermoFisher Scientific, USA). The mitochondria were lysed with RIPA buffer to extract the proteins. The cytosolic and mitochondrial proteins were adjusted to the same concentration. For Western blotting of cytochrome C (2000:1, antirabbit polyclonal, Abcam, UK) and AIF (2000:1, antirabbit polyclonal, Abcam, UK), the protein (50 µg) were loaded into each well of a SDS‐PAGE gel.

*Immunoblot Assay for ER Stress in the Presence of a Caspase Inhibitor*: NCI‐H460 cells (500 000 cells per well in a 6‐well plate) were incubated without and with AIP2 (0.25 × 10^−6^ m) for 1 d. For ER stress in the presence of a caspase inhibitor, the cells were pre‐treated with ZVAD‐FMK (40 × 10^−6^ m) for 1 h. The proteins (50 µg) were transferred to a PVDF membrane after electrophoresis. The corresponding PVDF membrane was treated with ER stress‐mediated primary antibodies (ATF‐6, ATF‐4, caspase‐12, IRE1α, p‐IRE1α, PERK, p‐PERK, CHOP, elF2α, p‐elF2α, JNK, and p‐JNK). HPR‐conjugated secondary antibody solution was added to the corresponding PVDF membranes before the overnight incubation at 4 °C. The immunoblot signals were visualized with an ECL reagent.

*Caspase‐3 Activity*: NCI‐H460 cells (500 000 cells per well in a 6‐well plate) were treated with the AIP series (0.25 × 10^−6^ m) for 1 d before the cells were lysed with RIPA buffer to extract the proteins. For the caspase activity in the presence of a caspase inhibitor, the cells were pretreated with ZVAD‐FMK (40 × 10^−6^ m, Santa Cruz Biotech, USA) for 1 h before the addition of the AIP series (0.25 × 10^−6^ m). The cells were lysed with RIPA buffer to extract the proteins, and all the protein sample concentrations were adjusted to 1.5 mg mL^−1^ using a BCA kit. The caspase activity was quantified by the addition of DEVD‐p‐NA (200 × 10^−6^ m) (Abcam, UK) prior to the detection of the absorbance at 405 nm using a microplate reader (Multiskan GO Microplate Spectrometer, ThermoFisher Scientific, USA).

*Animal Experiments*: All the experimental procedures involving animal studies were performed in accordance with the NIH Guideline for the Care and Use of Laboratory Animals. These procedures were approved by the Institutional Animal Care and Use Committee of Hanyang University.

*Antitumor Effects*: NCI‐H460 cells (5 × 10^6^ cells) were subcutaneously injected into 6‐week‐old male balb/c nude mice (*n* = 6). When the tumor volume became 50 mm^3^, HEPES buffer and AIPs (2 mg kg^−1^) were administrated by tail vein injection in the tumor bearing mice once every two days until day 11 (number of injection: 5 in each group). The tumor volume was measured using a caliper every 2 d and calculated with the following equation: Volume = Length × Width^2^ × 0.523. All the mice in the HEPES groups were sacrificed when the tumor volume exceeded 2500 mm^3^. The tumor growth and body weight of all the groups were monitored until day 23. In the following step, all the mice were sacrificed before the major organs (Lung, heart, kidney, spleen, and liver) were harvested to evaluate the cytotoxicity. All the organs were fixed with 10% formalin, embedded in paraffin, and cut into 5 µm thick sections. Representative sections were soaked in H&E and visualized by ZEISS optical microscopy.

*Immunohistochemical Assays*: Tumor xenografts were prepared as described above (*n* = 2). When the tumor size reached 150 mm^3^, HEPES buffer and AIPs (2 mg kg^−1^) were intravenously injected every two days. The total number of injections was 5. Two days after the final injection, the tumor tissues were excised and then fixed by 10% formalin solution. The preparation of the tumor sections was done as described above. The tumor sections were stained with H&E and terminal deoxynucleotidyl transferase dUTP nick‐end labeling (TUNEL). TUNEL assay was conducted according to the manufacturer's instructions (Merck, Darmstadt, Germany). For immunohistochemistry staining, the tumor sections were treated with the corresponding primary antibodies, cleaved caspase‐3 (antirabbit polyclonal, Abcam, UK), CHOP (antirabbit polyclonal, Abcam, UK), and CD31 (antirabbit polyclonal, Abcam, UK), and then incubated at room temperature for 20 min. with the Dako Envision Kit (Dako, Glostrup, Denmark) as a secondary antibody. Diaminobenzidine/hydrogen peroxidase (Dako) was used as the chromogen substrate. All slides were counterstained with Meyer's hematoxylin. For immunofluorescence, sections were treated with AIF (Abcam, antirabbit polyclonal, Abcam, UK) and cytochrome C (Abcam, antirabbit polyclonal, Abcam, UK), prior to the addition of the secondary antibody Fluor 488‐conjugated antirabbit IgG (Invitrogen, Carlsbad, CA, USA). Nuclear staining with 4,6‐diamidino‐2‐phenylindole (DAPI, Sigma‐Aldrich) and mitochondrial staining with MitoTracker Red (Invitrogen) were also performed. All the immunohistochemical images were obtained by ZEISS optical microscopy.

*Western Blot In Vivo*: Two days after the final injection, the excised tumor tissues were lysed with RIPA buffer and then homogenized. The preparation of the protein samples and Western blotting procedures were done as described in the “Western blotting in vitro section.” The same antibodies as in the “in vitro Western blotting” were used excluding caspase‐3 (1000:1, antirabbit polyclonal, Santa Cruz biotech, USA).

*Statistical Analysis*: Statistical significances were determined by a two‐tailed Student's *t*‐test. A *p* value < 0.05 was regarded as statistical significant.

## Conflict of Interest

The authors declare no conflict of interest.

## Supporting information

Supplementary
