# Supplementary material for: A Helical Polypeptide‐Based Potassium Ionophore Induces Endoplasmic Reticulum Stress‐Mediated Apoptosis by Perturbing Ion Homeostasis
Source: Adv Sci (Weinh). 2019 May 24;6(14):1801995. doi: 10.1002/advs.201801995 (PMC6661937; doi:10.1002/advs.201801995)
Supplement: Supplementary file 1 — Supplementary [file ADVS-6-1801995-s001.pdf]

Copyright WILEY-VCH Verlag GmbH & Co. KGaA, 69469 Weinheim, Germany, 2019.

ADVANCED SCIENCE
Open Access

Supporting Information

for *Adv. Sci.,* DOI: 10.1002/advs.201801995

A Helical Polypeptide-Based Potassium Ionophore Induces Endoplasmic Reticulum Stress-Mediated Apoptosis by Perturbing Ion Homeostasis

DaeYong Lee, Soo-Hwan Lee, Ilkoo Noh, Eonju Oh, Hyunil Ryu, JongHoon Ha, SeongDong Jeong, Jisang Yoo, Tae-Joon Jeon, Chae-Ok Yun, and Yeu-Chun Kim**

Supplementary information

Helical polypeptide-based potassium ionophore inducing ER stress-mediated apoptosis by perturbing ion homeostasis

DaeYong Lee[1+], Soo-Hwan Lee[2+], Ilkoo Noh[1], Eonju Oh[2], Hyunil Ryu[3], JongHoon Ha[1], SeongDong Jeong[1], Jisang Yoo[1], Tae-Joon Jeon[3], Chae-Ok Yun[2*], Yeu-Chun Kim[1*]

[1]Department of Chemical and Biomolecular Engineering, Korea Advanced Institute of Science and Technology (KAIST), Daejeon, Republic of Korea
[2]Department of Bioengineering, College of Engineering, Hanyang University, Seoul, Republic of Korea
[3]Deparment of Biological Engineering, Inha University, Incheon, Republic of Korea

[+]*These authors equally contributed to this work.*

Correspondence and requests for materials should be addressed to Prof. C.-O Yun (Email: chaeok@hanyang.ac.kr) or Prof. Y.C. Kim (Email: dohnanyi@kaist.ac.kr).

Experimental procedures

I. Physical characterization

All the NMR spectra were measured by a Bruker NMR spectrometer (400 MHz 54 mm NMR DD2, Agilent, USA). Deuterated dimethylsulfoxide (DMSO-d_6) and water (D_2O) were used as a NMR solvent. NCA (*N*-carboxyanhydride) formation and polymerization were conducted in a glove box (KK-011 AS, Korea Kiyeon, Republic of Korea). The molecular weight was obtained by gel permeation chromatography with a Younglin YL9100 HPLC system consisting of a quaternary pump, refractive index detector, adiabatic oven, vacuum degasser, and 10^3~10^5 g/mol *N*,*N*'-dimethylformamide (DMF) GPC column (KD-803, Shodex, Japan). 0.01 M LiBr DMF solution was used as the mobile phase at 35 °C with a 1 mL/min flow rate. The size and zeta potential were investigated by dynamic light scattering (DLS; ZetasizerNano ZD90, Malvern, UK). The sample concentration was adjusted to 1 mg/mL in HEPES buffer. The critical micelle concentration (CMC) of AIPs was determined by Nile Red as a fluorescence probe. Nile Red (5 μL in 0.5 mg/mL acetone) was added in AIPs solutions preparing from 0.1172 to 1.5 mg/mL in deionized water. Each solution was stirred at RT until acetone was evaporated. Each mean fluorescence intensity was measured by a fluorescence spectrometer (λ_{ex} = 526 nm, λ_{em} = 636 nm; Gemini XPS microplate reader, MOLECULAR DEVICES, USA).

II. Synthesis

Poly(N^{ε}-Cbz-L-lysine-*random*-N^{ε}-TFA-L-lysine)

N^{ε}-Cbz-L-lysine *N*-carboxyanhydride (NCA) and N^{ε}-TFA-L-lysine NCA were prepared as previously described in the literature. In a glove box, N^{ε}-TFA-L-lysine NCA (1.75 g, 6.53 mmol) and N^{ε}-Cbz-L-lysine NCA (3.00 g, 9.79 mmol) were dissolved in anhydrous DMF (20 mL) with hexamethyldisilazane (22.6 μL, 0.108 mmol) and diisopropylethylamine (50 μL). The polymerization was carried out at RT for 48 h. The viscous solution was precipitated with excessive deionized water, and the white powder was washed with deionized water three times. The white powder was freeze-dried to obtain poly(N^{ε}-Cbz-L-lysine-*random*-N^{ε}-TFA-L-lysine) (3 g). The repeating units and its polydispersity index were approximately 150 and 1.07, respectively, as determined by GPC. Cbz-lysine and TFA-lysine were almost 60 and 90, respectively, as characterized by NMR spectroscopy (Figure S2).

Poly(N^{ε}-Cbz-L-lysine-*random*-L-lysine)

TFA protecting groups were removed under mild basic conditions using potassium carbonate. TFA groups were completely removed, determined by NMR spectroscopy (Figure S3).

Poly(N^{ε}-Cbz-L-lysine-*random*-4-hydroxybenzoyl-L-lysine)

4-acetoxybenzoyl chloride was prepared with a slight modification of a previously reported procedure. Poly(N^{ε}-Cbz-L-lysine-*random*-L-lysine) (1.00 g) was dissolved in anhydrous DMF (10 mL) with trimethylamine (6 equivalents of lysine residues) and 4-acetoxybenzoyl chloride (5 equivalents of lysine residues). The reaction mixture was stirred at RT overnight. To remove the unreacted reagents and isolate the intermediate product, the mixture was precipitated with excessive saturated sodium carbonate solution. A brownish solid was isolated by vacuum filtration and washed with deionized water three times. The brownish solid was suspended in 1 N NaOH solution to deprotect the ester group. The transesterification was done at RT overnight. The cloudy solution was slowly treated with 35 wt% HCl solution until a precipitate was observed. A brownish powder was isolated by filtration and washed with deionized water three times. The brownish powder was lyophilized to obtain poly(N^{ε}-Cbz-L-lysine-*random*-4-hydroxybenzoyl-L-lysine) (0.93 g). The degree of modification was more than 90%, as evaluated by NMR spectroscopy. (Figure S4)

Poly(N^{ε}-Cbz-L-lysine-*random*-4-(5-bromopentoxy)benzoyl-L-lysine)

Poly(N^{ε}-Cbz-L-lysine-*random*-4-hydroxybenzoyl-L-lysine) (0.6 g) was dissolved in anhydrous DMF (10 mL) with potassium carbonate (0.96 g, 70 mmol) and 1,5-dibromopentane (15 equivalents of the phenol moiety). The reaction mixture was stirred at 90 °C for 1 day. The mixture was precipitated with diethyl ether and washed three times to completely remove the excessive unreacted reagent. The crude product was washed with deionized water to remove the salt before the lyophilization. Poly(N^{ε}-Cbz-L-lysine-*random*-4-(5-bromopentoxy)benzoyl-L-lysine) was obtained. The degree of modification was almost 100% due to the disappearance of the peak (–OH of phenol) (Figure S5).

Poly(N^{ε}-Cbz-L-lysine-*random*-4-(1-aza-18-crown-6 ether pentoxy)benzoyl-L-lysine-*random*-4-(5-trimethylammoniumpentoxy)benzoyl-L-lysine)

In the first step, 1-aza-18-crown-6 ether (1 equivalent of the bromide groups) was added to DMF solution (10 mL) in which poly(N^{ε}-Cbz-L-lysine-*random*-4-(5-bromopentoxy)benzoyl-L-lysine) was solubilized. The substitution reaction was carried out at 90 °C for 1 day. Thereafter, trimethyl amine solution (3 equivalents of the bromide groups, 2 M in acetonitrile) with sodium iodide (3 equivalents of the bromide groups) were added to form the trimethyl ammonium moiety. The reaction mixture was precipitated with saturated sodium carbonate

solution, and a yellowish precipitate was isolated by centrifugation. The powder was washed with deionized water and then lyophilized to obtain Poly(N^{ε}-Cbz-L-lysine-*random*-4-(1-aza-18-crown-6 ether pentoxy)benzoyl-L-lysine-*random*-4-(5-trimethylammoniumpentoxy)benzoyl-L-lysine). The total degree of modification was 71.3% (35.1% of 1-aza-18-crown-6, 36.2% of trimethylammonium moiety), quantified by NMR spectroscopy (Figure S6).

Poly(L-lysine-*random*-4-(1-aza-18-crown-6 ether pentoxy)benzoyl-L-lysine-*random*-4-(5-trimethylammoniumpentoxy)benzoyl-L-lysine) (AIP)

N^{ε}-Cbz protecting groups were deprotected by acidolysis using HBr to obtain AIP. The peaks indicating the benzyl positions were completely removed (100% deprotection). (Figure S7)

Amidation

Prior to preparing the amidating reagent, carboimmide chemistry was used to convert the carboxylic acid group to the NHS ester group. Briefly, acetic acid, butyric acid, hexanoic acid, or octanoic acid solubilized in DMF (5 mL) was converted to the *N*-hydroxysuccinimide (NHS) ester form using 1-(3-dimethylaminopropyl)-3-ethylcarbodiimide (EDC) HCl (3 equivalents of carboxylic acid groups) and NHS (2 equivalents of carboxylic acid groups). The corresponding R-NHS ester (3 equivalents of the lysine residues) was solubilized with TEA which was slowly poured into the AIP solution (2 mL, 1.2 mg/mL in deionized water). The amidation reaction was carried out at RT for 1 day. The corresponding product was isolated by centrifugation and then washed with deionized water three times. The polypeptide was treated with 0.1 N HCl (100 μL) to protonate the product and then dialyzed against deionized water to remove the excessive HCl before lyophilization.

FITC-labelling

AIP (0.2 g) was solubilized in PBS (5 mL) with FITC (3 mL, 0.7 mg/mL in DMSO, Tokyo Chemical Industry, Co., LTD., Japan), and then, the reaction mixture was stirred at RT for 1 day. The unreacted FITC and DMSO were removed by a dialysis method (Molecular weight cutoff: 3500 g/mol, SPECTRUMLABS, USA) before the solution was freeze-dried to obtain FITC-labelled AIP. The FITC-labelled AIP series were synthesized with the method described above.

IR800CW-labelling

AIP (0.2 g) was solubilized in PBS (5 mL) with IR800CW (100 μL, 50 mg/mL, LI·COR, USA) and 1-(3-Dimethylaminopropyl)-3-ethylcarbodiimide (EDC) HCl (19 mg, 100 μmol) and NHS (8 mg, 69 μmol). The

reaction was carried out at RT for 1 day. The unreacted species were removed by a dialysis method for 1 day. The solution was lyophilized to obtain IR800CW-tagged AIP. The IR800CW-tagged AIP series were prepared by the method described above.

III. CD characterization

CD spectrometry with different potassium levels

All the AIP samples were solubilized in various concentrations of KCl solutions (0.15, 1.5, 15 and 150 mM). The final concentration was adjusted to 1 mg/mL. CD spectrometry was performed by the same procedure described in the manuscript.

LUV preparation

In a 100 mL dry round bottom flask, EYPC (20 mg/mL in chloroform, Sigma Aldrich, USA) was dried by a rotary evaporator at RT to form a thin phospholipid film.[1] To completely remove the chloroform, the thin film was evaporated under reduced pressure overnight before the buffer (1 mL in 10 mM HEPES and 100 mM KCl) was added while a magnetic bar was stirred at 1000 rpm for 1 h. The lipid solution was rapidly frozen in liquid nitrogen and then melted at RT. The freeze-thaw cycle was repeated 12 times. Extrusion was carried out 19 times by a mini extruder with a polycarbonate membrane (pore size 200 nm, Avanti).

AIP-Lipid CD spectrometry

Prior to the CD measurement, LUVs were prepared in HEPES-buffered solution (10 mM HEPES, 50 mM NaCl, 50 mM KCl). The AIPs solubilized in HEPES-buffered solution were mixed with the LUVs at different molar ratios (100;1, 50:1, 25:1 and 10:1). CD spectrometry was performed by the same procedure described in the manuscript.

IV. Ion transport experiments

HPTS-loaded LUV preparation

A thin phospholipid film was hydrated with HEPES-buffered solution (1 mL in 1 mM HPTS (Sigma Aldrich, USA), 100 mM MCl, and 10 mM HEPES).[1] The free-thaw cycles were repeated 10 times before lipid vesicles were extruded using a mini extruder (200 nm polycarbonate filter). The encapsulated HPTS was removed by a dialysis method in HEPES buffered solution (10 mM HEPES, 100 mM MCl). HPTS-loaded LUV solution (100 μL) was diluted to HEPES-buffered solution (10 mL in 10 mM HEPES, 100 mM MCl). (M; Li, Na, K, or Cs)

HPTS assay in a concentration-dependent manner

HPTS-loaded LUV solution (100 μL in 10 mM HEPES, 100 mM KCl) was added to a well (96 well plate). Treatment with 0.1 N KOH solution (1 μL) was done to form a pH-gradient between the outside and inside of the lipid vesicles. After 30 sec., the AIP series (1, 0.75, 0.5, 0.25 and 0.125 μM at final concentrations in 10 mM HEPES, 100 mM KCl) were added, and then, the solution was pipetted four times for mixing. Time-dependent fluorescence intensity was monitored every second until 200 sec. before the lipid vesicle was lysed by 0.1% Triton X-100 (10 μL) to obtain the saturated fluorescence intensity (λ_{ex} = 450 nm, λ_{em} = 510 nm, Gemini XPS microplate reader, MOLECULAR DEVICES, USA). Normalized fluorescence intensity (I_F) was obtained by the following equation: $(F_t - F_0)/(F_s - F_0)$ X 100 (F_t; fluorescence intensity at time t, F_0; fluorescence intensity at 0 sec, F_s; Saturated fluorescence intensity after the addition of 0.1% triton-X100).

Cation selectivity

HPTS-loaded LUV solution (100 μL in 10 mM HEPES, 100 mM MCl, M; Li, Na, K, and Cs) was added to a well (96 well plate). The corresponding treatment of 0.5 N MOH solution (2 μL) was done to form a pH-gradient between the outside and inside of the lipid vesicles. Time-dependent fluorescence intensity was monitored every second until 200 sec. prior to the lysis of the lipid vesicle using a 0.1% triton-X100 solution. Each I_F was obtained by the procedure described above.

Anion selectivity

HPTS-loaded LUV solution (100 μL in 10 mM HEPES, 100 mM KX, X; F, Cl, Br, and I) was added to a well (96 well plate). 0.5 N KOH solution (2 μL) was added to each well. Time-dependent fluorescence intensity was measured every second until 200 sec. before the lysis of the LUVs using a 0.1% triton-X100 solution. Each I_F was obtained by the procedure described above.

Single channel analysis

A planar lipid bilayer membrane was prepared as in our previous work [2,3]. In this experiment, 3% DPhPC (1,2-diphytanoyl-sn-glycero-3-phosphocholine, Avanti Polar Lipids, Alabaster, AL, USA) dissolved in n-decane (MP Biomedicals, Irvine, CA, USA) was used to form a lipid bilayer on an 50-80 μm aperture in a 10-μm-thick PTFE film (Goodfellow, Huntingdon, UK). The aperture was fabricated using a spark generator (DAEDALON, Salem, MA, USA). The lipid solution was spread around the aperture of the PTFE film and was then dried for 30 min. The PTFE film was placed between two chambers, and both chambers were filled with electrolyte solution (0.5 M KCl at pH 7.0). A planar lipid bilayer membrane was formed by spreading ~0.5 μL of DPhPC solution on the aperture of the PTFE film [4]. The Ag/AgCl electrodes were submerged in both chambers to measure the electrical current across the aperture, and 150 mV was applied. After the formation of a lipid bilayer was observed by a computer microscope (Digital Blue, QX5, Marietta, GA, U.S.A), the synthesized compounds (a,b,c,d) were dispensed into both sides of the chamber up to 1 μM, and the current traces were observed over 30 min. Then, gramicidin A (Sigma, St. Louis, MO, U.S.A) to a final concentration of 1ng/mL was added to confirm the presence of a lipid bilayer. The currents across the membrane were recorded using an Axopatch 200B amplifier and Digidata 1440A data acquisition system (Molecular Devices, Sunnyvale, CA, U.S.A). Currents sampled at 250 kHz with a low-pass Bessel filter (1 kHz). Data were collected using the Clampfit 10.3 software and further analyzed using the Clampex 10.3 software (Molecular Devices, Sunnyvale, CA, U.S.A).

V. *In vitro* experiments

Demonstration of AIPs binding to cell membranes

NCI-H460 cells (15000 cells/well in a 12 well plate) were treated with FITC-labelled AIPs (25 nM) for 3 h. The cells were rinsed with PBS three times and trypsinized. To prepare extracellular fluorescence-quenched cells, the isolated cells were incubated with trypan blue solution (2wt% in PBS) for 2 min, and washed with PBS three times. The cells were fixed with a 4% paraformaldehyde solution. Green fluorescence-positive cells were assessed by flow cytometry. The degree of AIPs binding to cell membranes was calculated by the following equation; MFI_{AIPs}-$MFI_{\mathrm{TB\text{-}treated\ AIPs.}}$ (TB: trypan blue).

Evaluation of Cytotoxicity

A methyl thiazolyl tetrazolium (MTT, Sigma Aldrich, USA) assay was used against NCI-H460 (human lung carcinoma, Korean Cell Line Bank, Korea), MDA-MB-231 (Human breast adenocarcinoma, Korean Cell Line Bank, Korea) and HT-1080 (Human fibrosarcoma, Korean Cell Line Bank, Korea) to confirm the cytotoxicity of the AIPs. The cells were seeded on 96 well plates at 10000 cells/well in Dulbecco Modified Eagle Medium (DMEM, Sigma Aldrich, USA) including 10% heat-inactivated fetal bovine serum and 1% antibiotic antimycotic solution and then incubated in a 5% CO_2 atmosphere at 37 °C for 1 day. The old medium was replaced with serum-free media containing various concentrations of the AIP solution (25, 2.5, 0.25, 0.025, 0.0025 and 0.00025 μM solubilized in 10 mM HEPES, 100 mM NaCl solution) prior to further incubation for 1 day. MTT solution (2.5 mg/mL in phosphate buffer saline (PBS), 20 μL) was added to each well, and then, the cells were incubated for 3 h before dimethylsulfoxide (DMSO) (100 μL) was added to solubilize the formazan. The UV-Vis absorbance (A) of all the wells was detected by an UV-Vis spectrometer (MultiSkan^{TM} Go, Thermo Scientific, USA) at 590 nm. The relative cell viability was obtained by the ratio of A_{sample} to A_{control}. Each IC_{50} value was obtained by logarithm regression.

Immunofluorescence staining of AIF

NCI-H460 cells (80000 cells/well in a 24 well plate) seeded onto a coverslip were treated with the AIP series (0.25 μM) for 24 h prior to fixation with a 4% paraformaldehyde solution. For the AIF activity in the presence of a caspase inhibitor, the cells were pre-treated with ZVAD-FMK (40 μM, Santa Cruz Biotech, USA) for 1 h before the addition of the AIP series (0.25 μM). The cells were blocked with 1 wt% BSA PBST for 1 h and then treated with the corresponding primary antibody solution (anti-rabbit AIF; 1000:1 in 1 wt% BSA PBST) at 4 °C overnight. Alexa fluor 488-tagged secondary antibody solution (1% BSA, 0.3% Triton X-100 in PBS, goat anti-rabbit; 1000:1)

was added to the wells and incubated for 2 h. In the last step, all the nuclei were stained with DAPI (300 nM in PBS) for 10 min.

Visualization of apoptotic nuclear fragmentation

NCI-H460 cells (80000 cells/well in a 24 well plate) seeded onto a coverslip for a 24 well plate were incubated with the AIP series (0.25 μM) for 12 h. The cells were fixed with 4% para-formaldehyde solution for 10 min. after washing with PBS three times. Thereafter, the cells were stained with DAPI (300 nM) for 10 min. Apoptotic nuclear fragmentation was visualized by CLSM.

VI. *In vivo* experiments

Biodistribution study

The formation of tumor xenografts were done as described above (n = 3). When the tumor volume reached approximately 150 mm^3, IR800CW-labelled AIPs (2 mg/kg) were intravenously injected into the mice. All the NIRF signals were detected at predetermined time points (1, 3, 6, 12, 24, and 48 h). After 2 days, all the organs in each group (tumor, heart, lung, liver, spleen and kidney) were excised, and the NIRF signals were analyzed by a Pearl® Impulse Small Animal Imaging System (LI-COR, Lincoln, NE).

Blood compatibility

Blood (1 mL) obtained from a mouse was centrifuged at 1200 g for 10 min. before the removal of the supernatant. Thereafter, the blood was treated with PBS (1 mL) and then centrifuged at 1200 g for 10 min. after washing with PBS twice. The isolated red blood cells (RBC) were diluted in a saline solution (10 mL). The RBCs (900 μL) were treated with HEPES buffer (100 μL), AIPs (100 μL, 0.2 mg/mL), and Triton X-100 (100 μL). The final AIP concentrations were adjusted to 20 μg/mL. Sample-treated RBCs were incubated at 37 °C for 1 h prior to isolating RBCs by centrifugation at 4000 g for 5 min. Each supernatant was transferred to a 96-well plate to detect the UV absorbance at 540 nm r (MultiSkan™ Go, Thermo Scientific, USA). Hemolytic activity (%) was calculated as follows: $(A_{\text{sample}}-A_{\text{untreated}})/(A_{\text{TritonX-100}}-A_{\text{untreated}})$ X 100 (%).

Assessment of *in vivo* toxicity

To assess the liver and kidney toxicity, serum samples were collected at 2 days post-injection, and the levels of alanine aminotransferase (ALT) and aspartate aminotransferase (AST), blood urea nitrogen (BUN), and creatinine were analyzed by Seegene (Seoul, Korea).

Immunoblotting of angiogenesis-related proteins *in vitro*

Electrophoresis was conducted by loading 50 μg of proteins into each well of SDS-PAGE gel before the proteins were transferred to a PVDF membrane (0.2 μm pore size PVDF membrane, Roche, Swiss). The PVDF membrane was treated with the primary antibody solutions (MMP-2; 1000:1, anti-rabbit polyclonal, Cell Signaling Technology, USA, MMP-9; 1000:1, Cell Signaling Technology, Abcam, USA, VEGF; 1000:1, anti-rabbit polyclonal, SantaCruz Biotech, USA). Thereafter, the PVDF membranes were treated with the secondary antibody solutions. The blot signals were visualized by an ECL reagent.

Supplementary figures

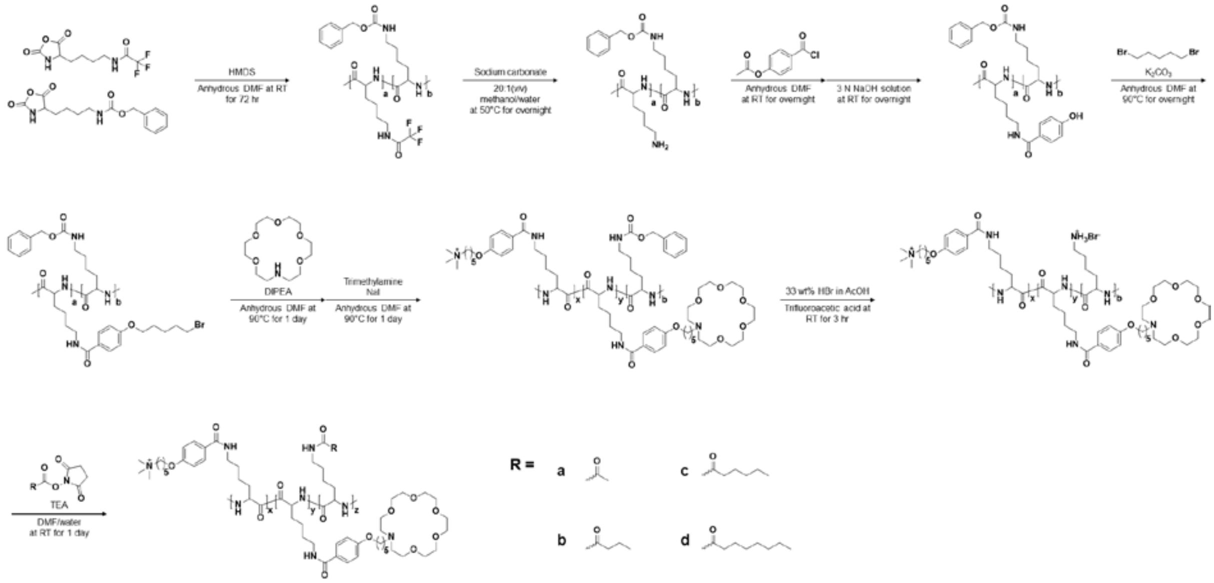

Figure S1. Synthetic scheme of AIP series.

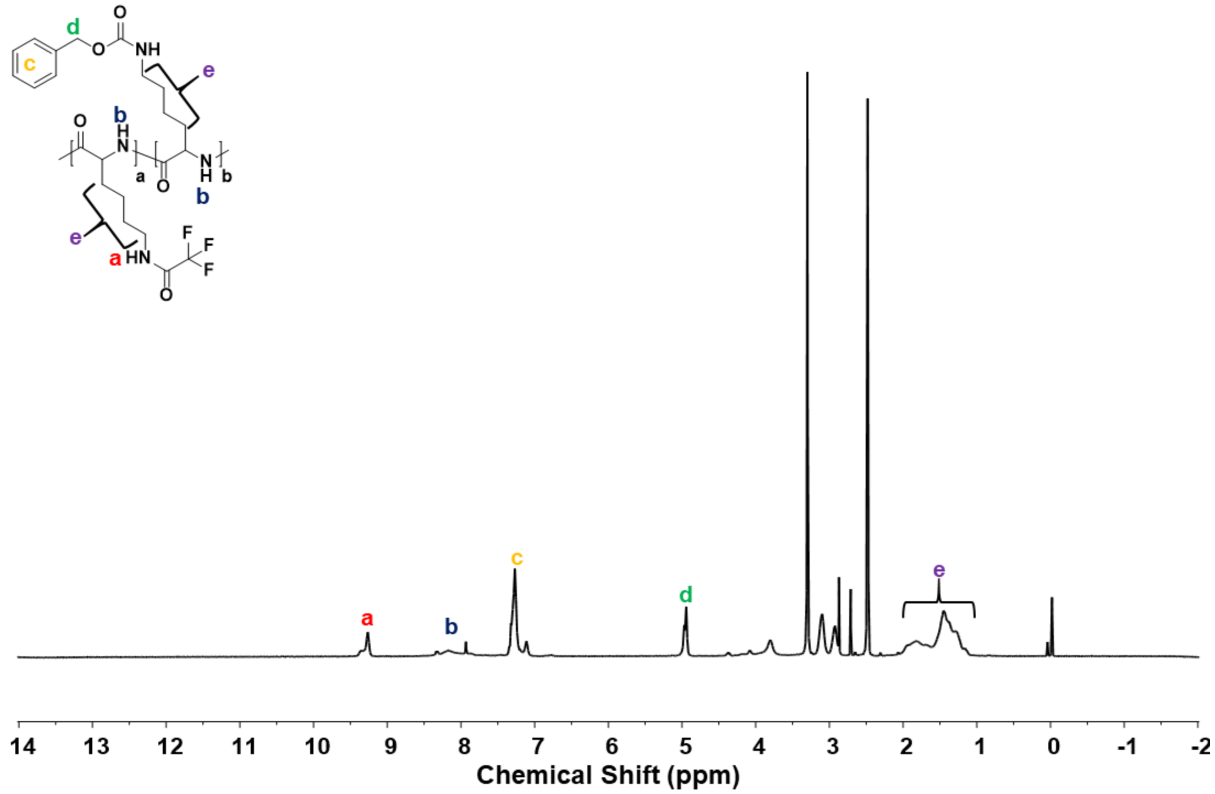

Figure S2. NMR spectrum of poly(N^{ε}-Cbz-L-lysine-*random*-N^{ε}-TFA-L-lysine) dissolved in DMSO-d_6.

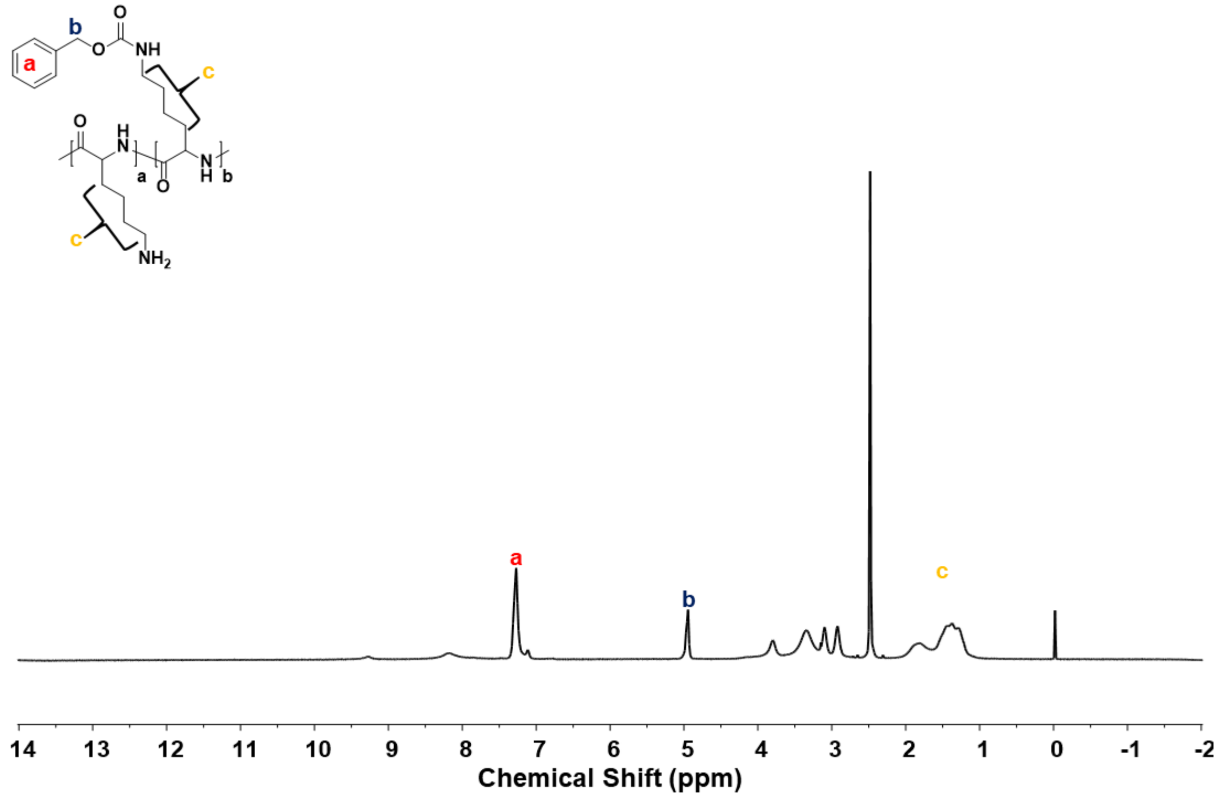

Figure S3. NMR spectrum of poly(N^{ε}-Cbz-L-lysine-*random*-L-lysine) dissolved in DMSO-d_6.

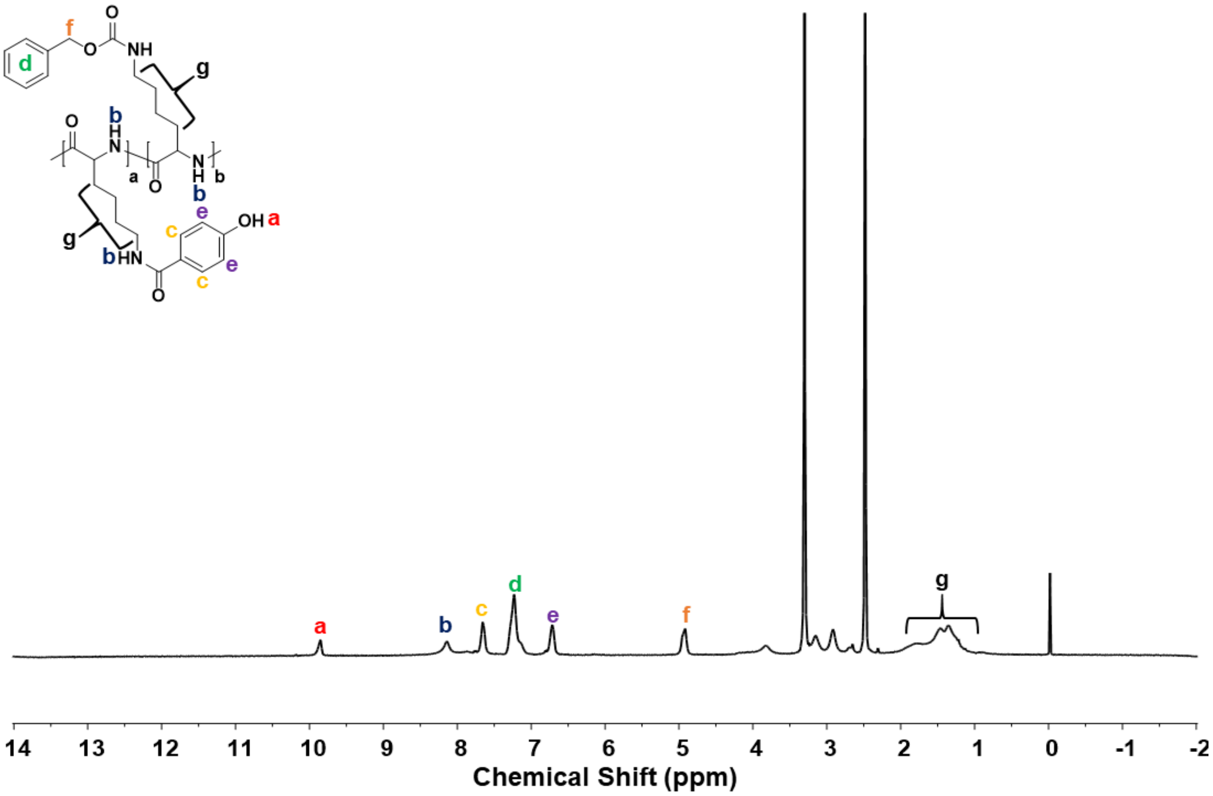

Figure S4. NMR spectrum of poly(N^{ε}-Cbz-L-lysine-*random*-4-hydroxybenzoyl-L-lysine) dissolved in DMSO-d_6.

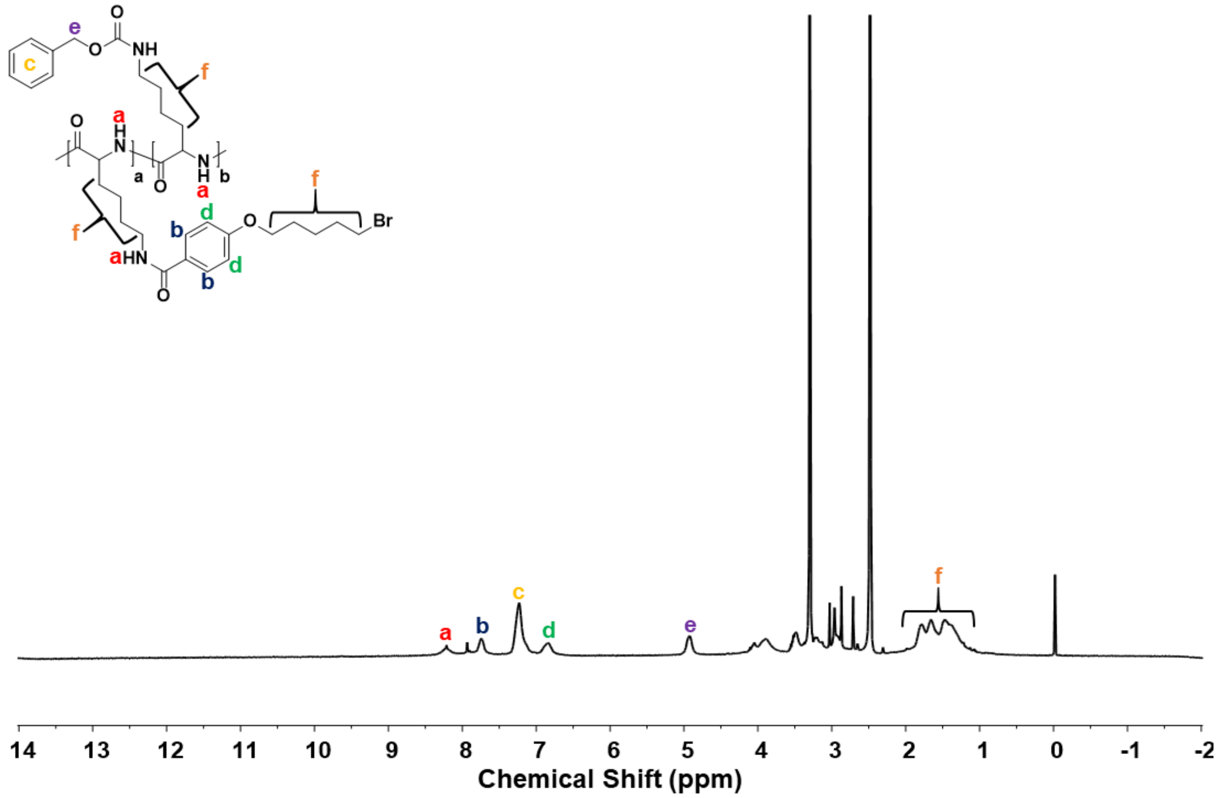

Figure S5. NMR spectrum of poly(N^{ε}-Cbz-L-lysine-*random*-4-(5-bromopentoxy)benzoyl-L-lysine) dissolved in DMSO-d_6.

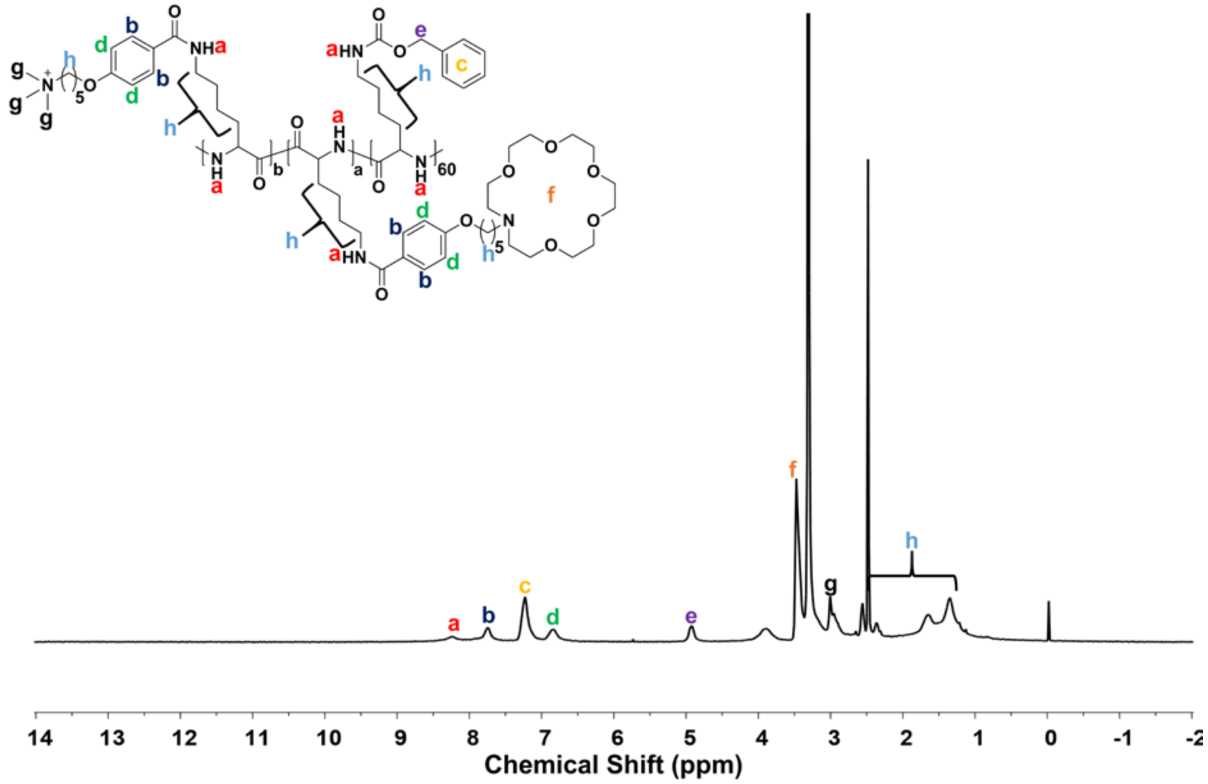

Figure S6. NMR spectrum of poly(N^{ε}-Cbz-L-lysine-*random*-4-(1-aza-18-crown-6 ether pentoxy)benzoyl-L-lysine-*random*-4-(5-trimethylammoniumpentoxy)benzoyl-L-lysine) in DMSO-d_6.

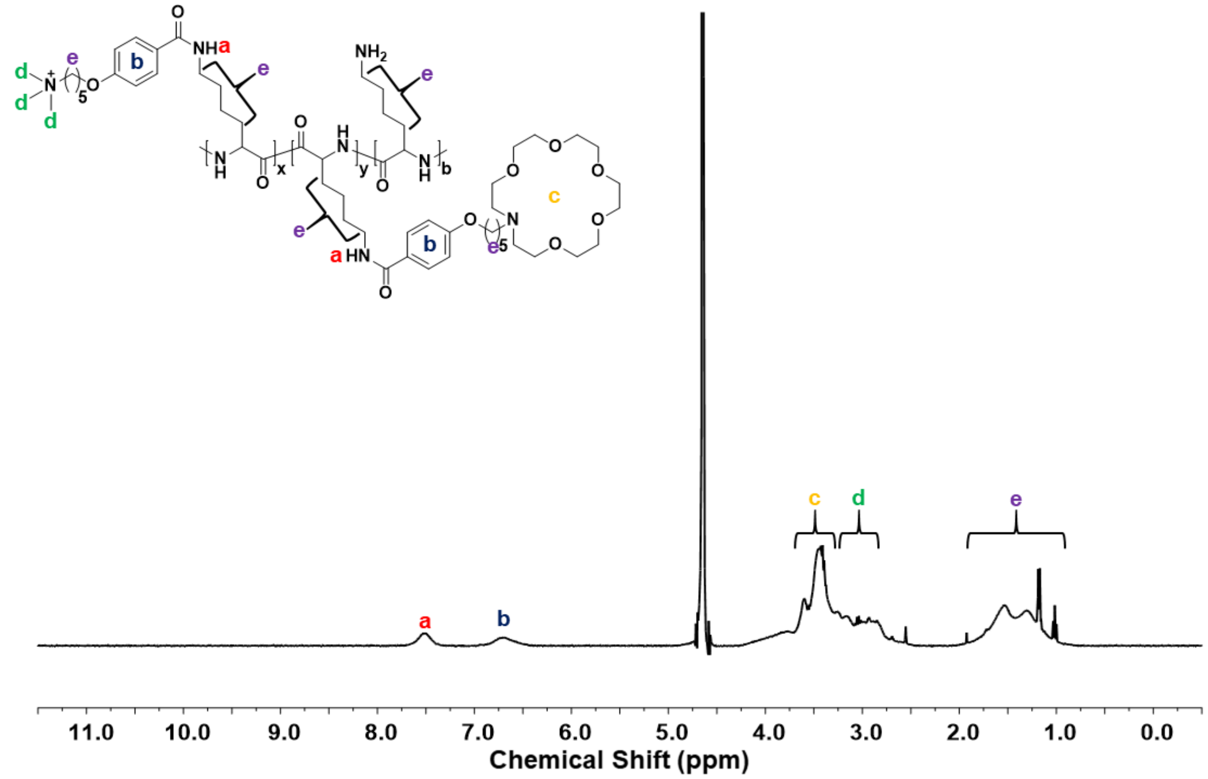

Figure S7. NMR spectrum of AIP in D_2O.

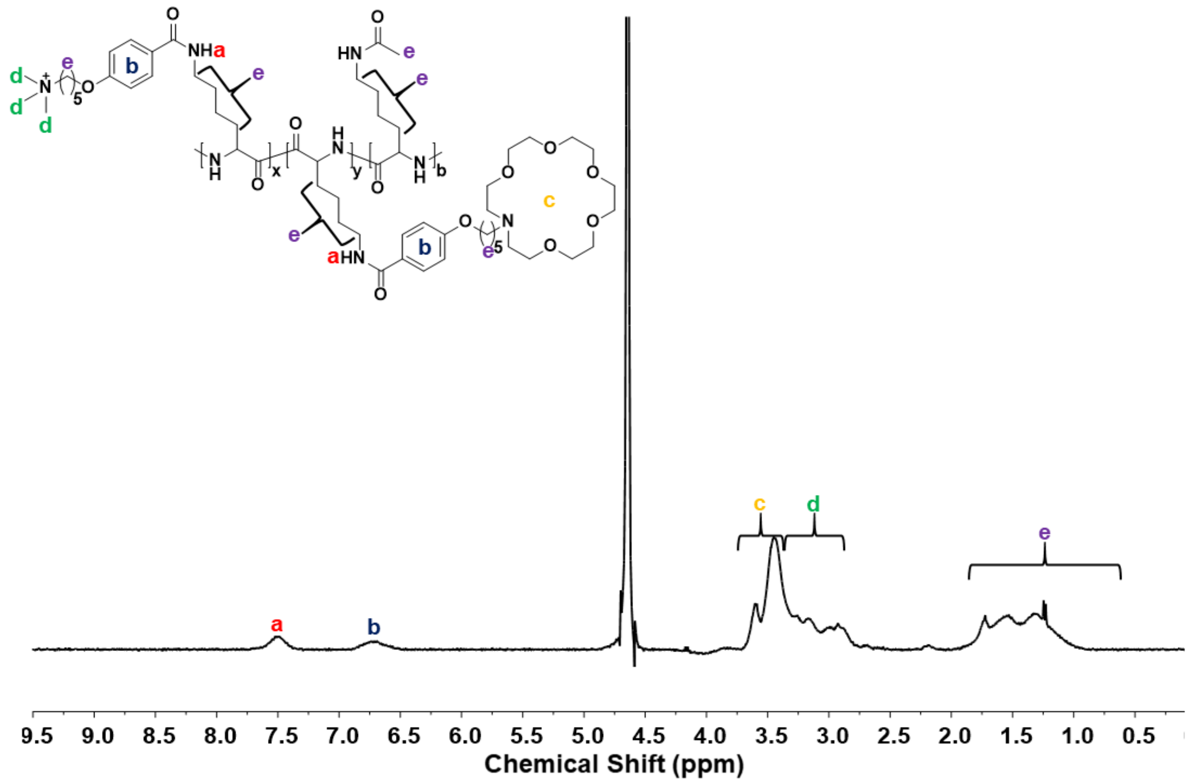

Figure S8. NMR spectrum of AIP1 in D_2O.

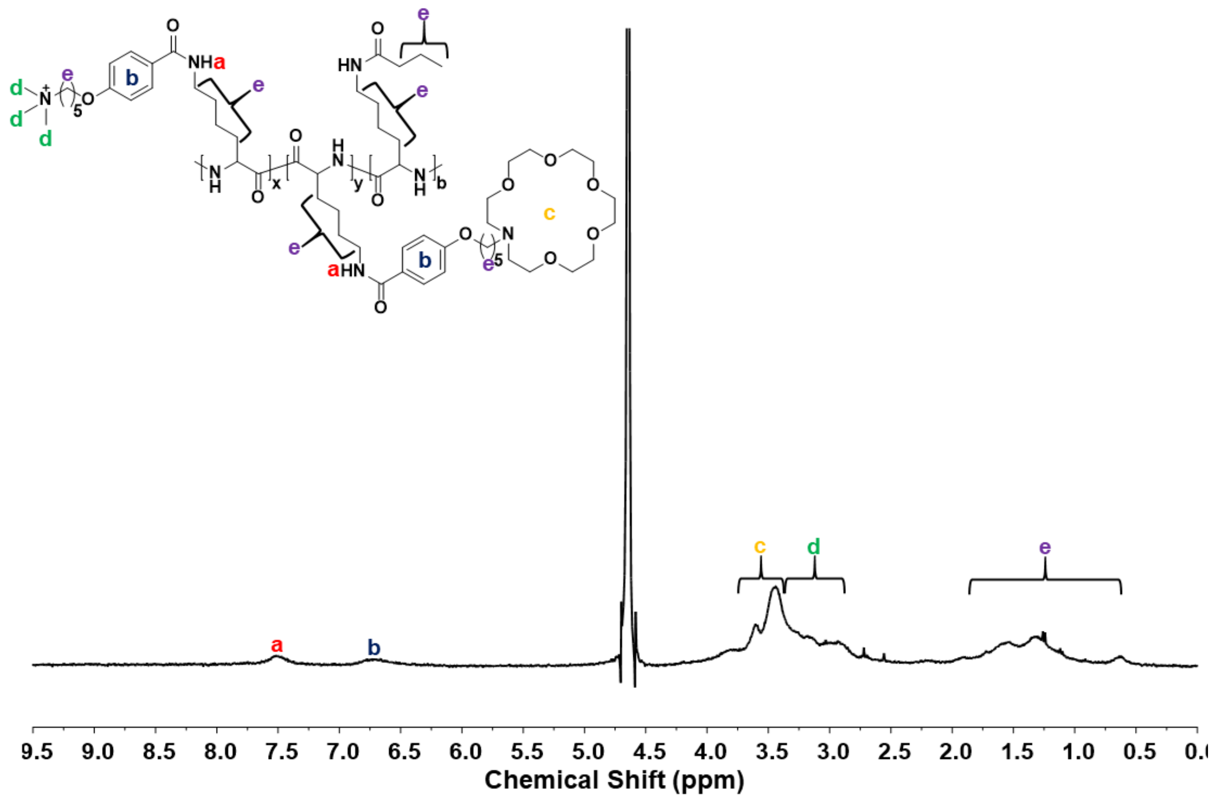

Figure S9. NMR spectrum of AIP2 in D_2O.

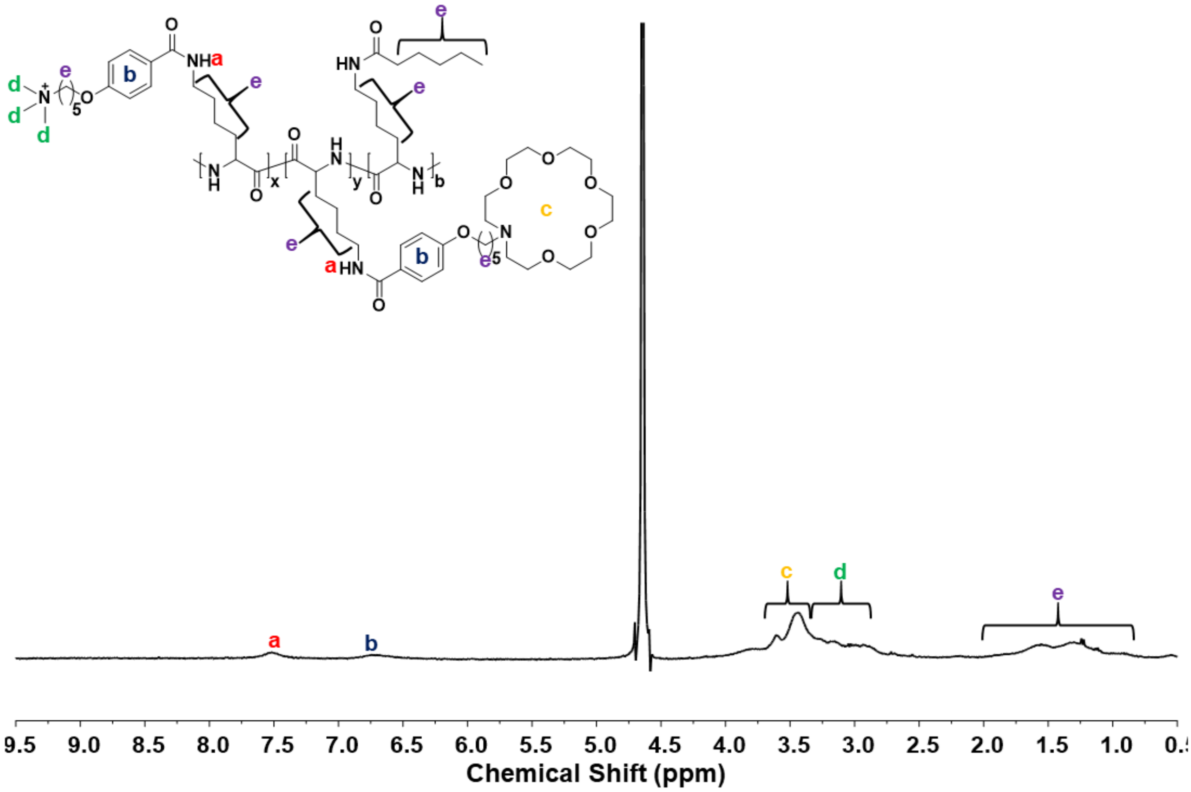

Figure S10. NMR spectrum of AIP3 in D_2O.

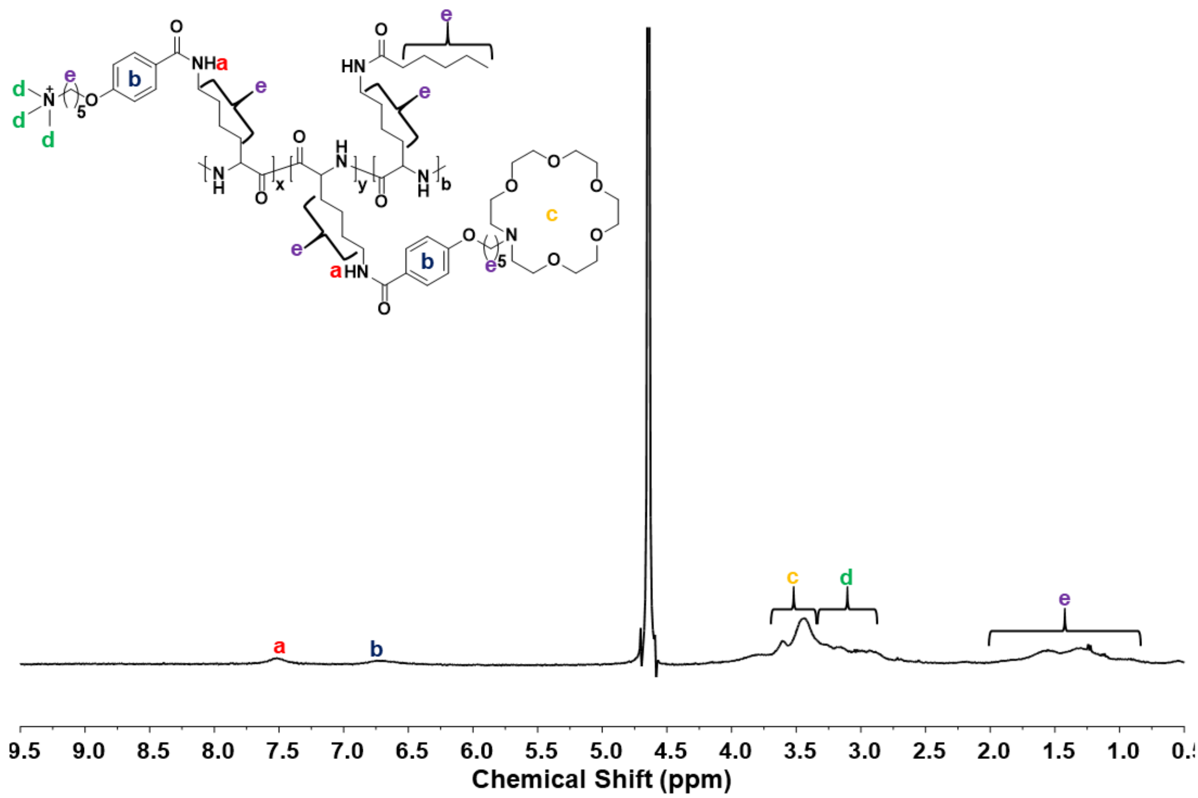

Figure S11. NMR spectrum of AIP4 in D_2O.

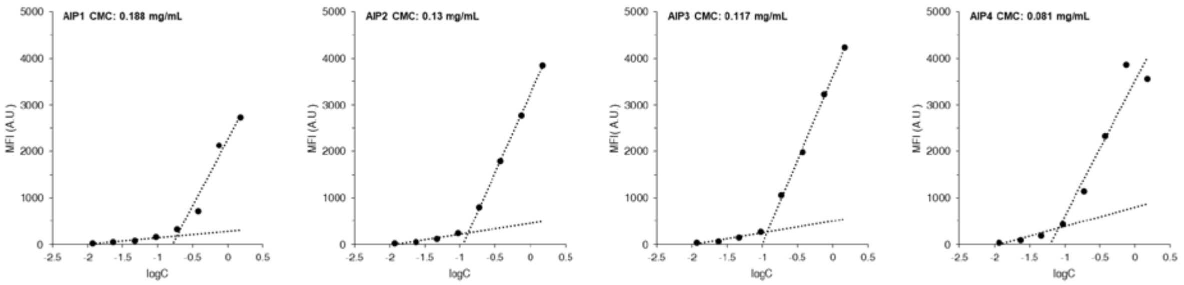

Figure S12. Determination of AIPs' CMC values. Each CMC value was measured based on the MFI.

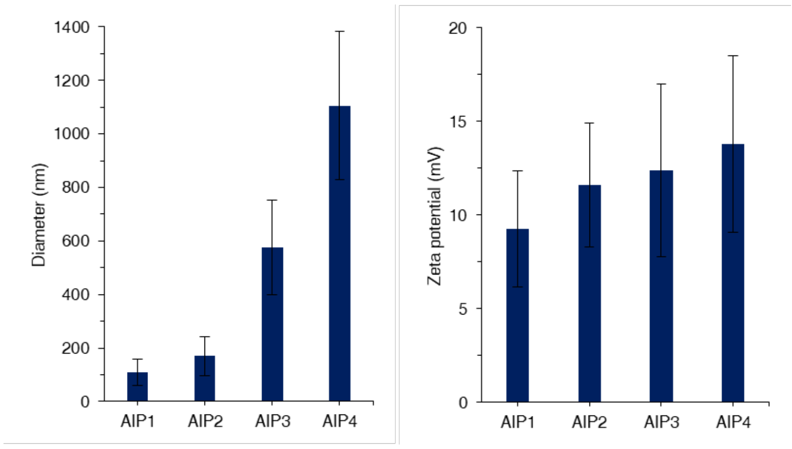

Figure S13. hydrodyanamic diameter and zeta potential of AIPs. AIPs were solubilized in HEPES buffer (1 mg/mL).

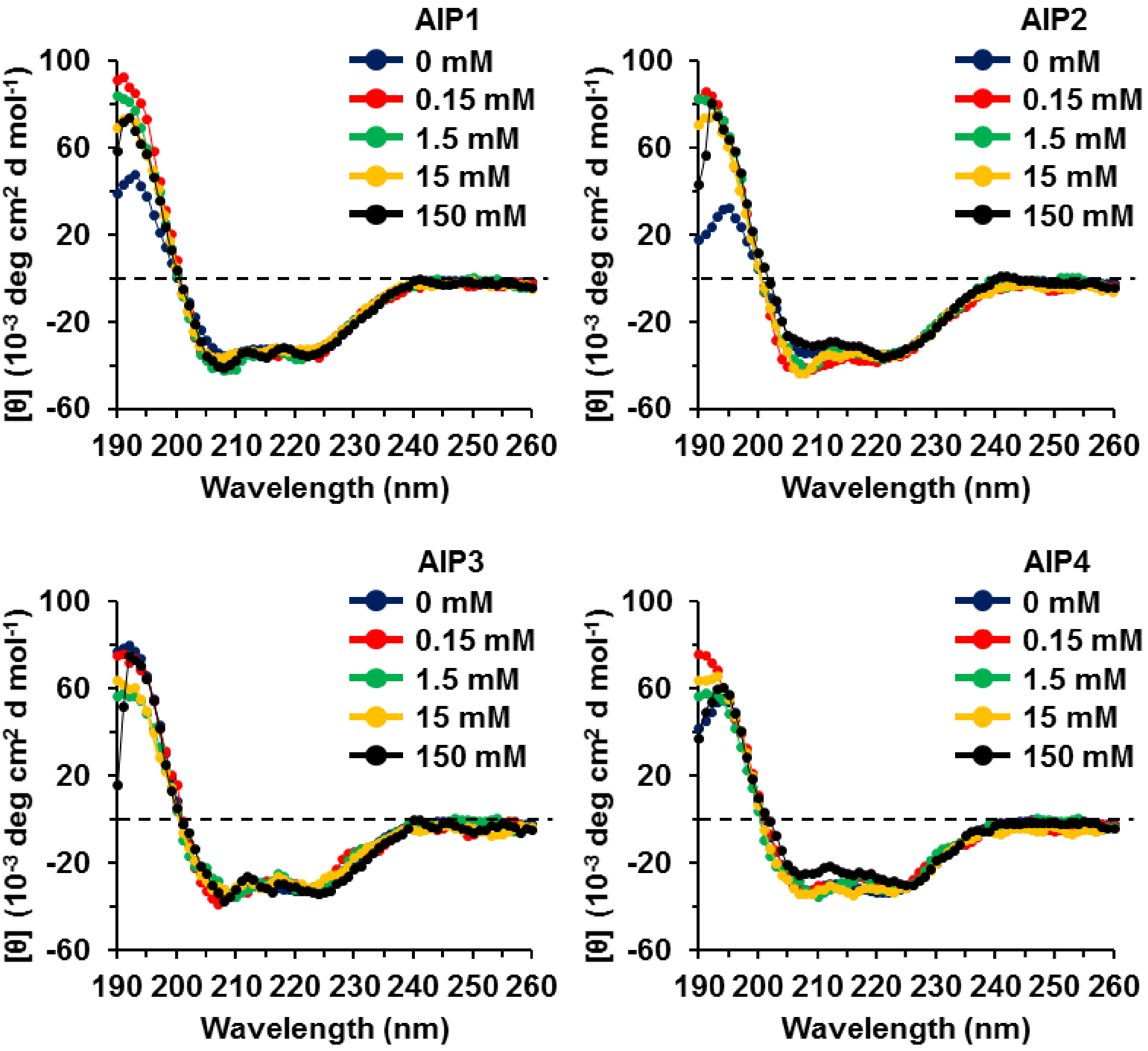

Figure S14. CD spectra of AIPs solubilized in different KCl aqueous solutions (0, 0.15, 1.5, 15 and 150 mM)

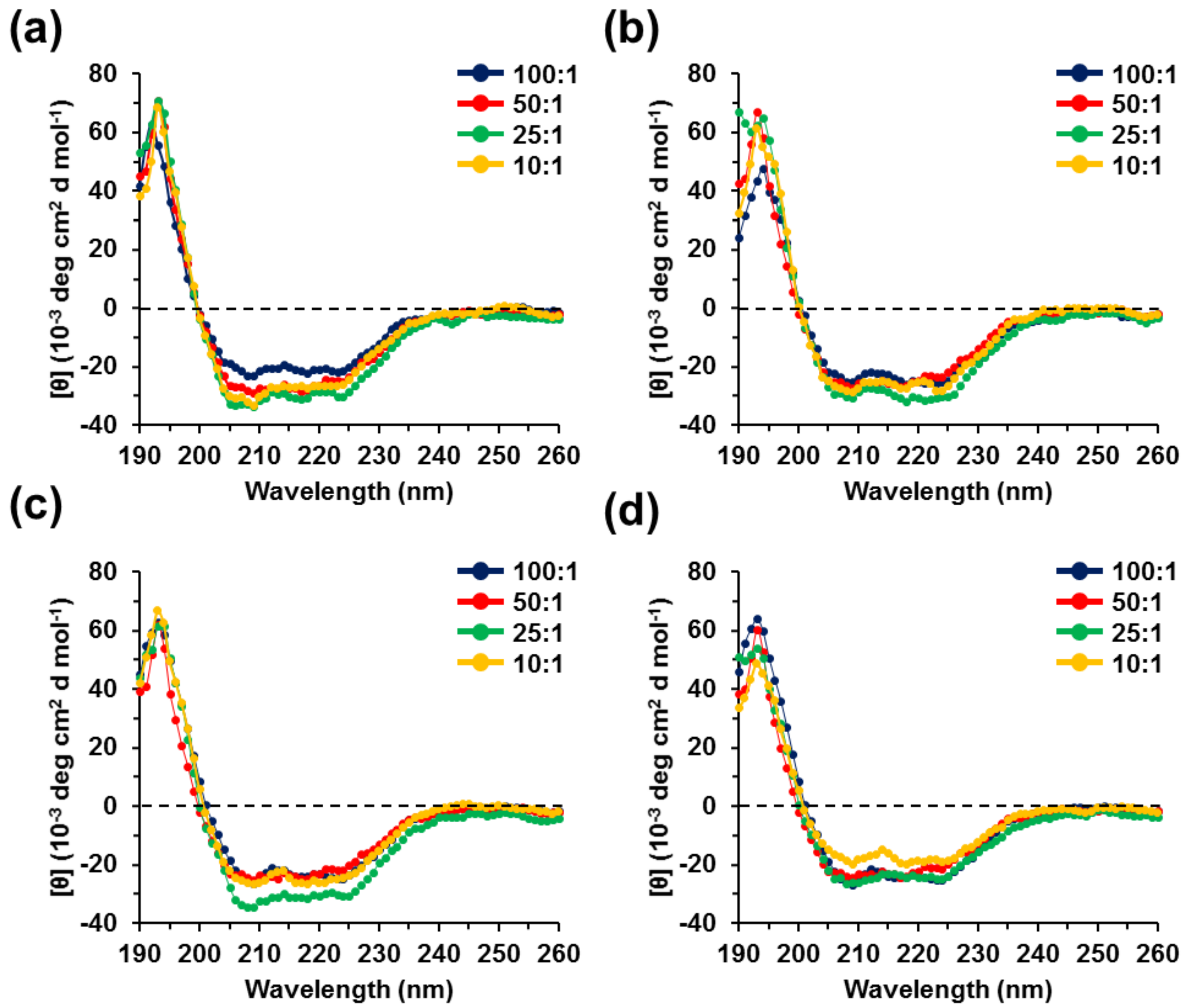

Figure S15. CD spectra of AIPs in the presence of LUVs in different molar ratios (100:1, 50:1, 20:1 and 10:1). (a) AIP1, (b) AIP2, (c) AIP3, and (d) AIP4

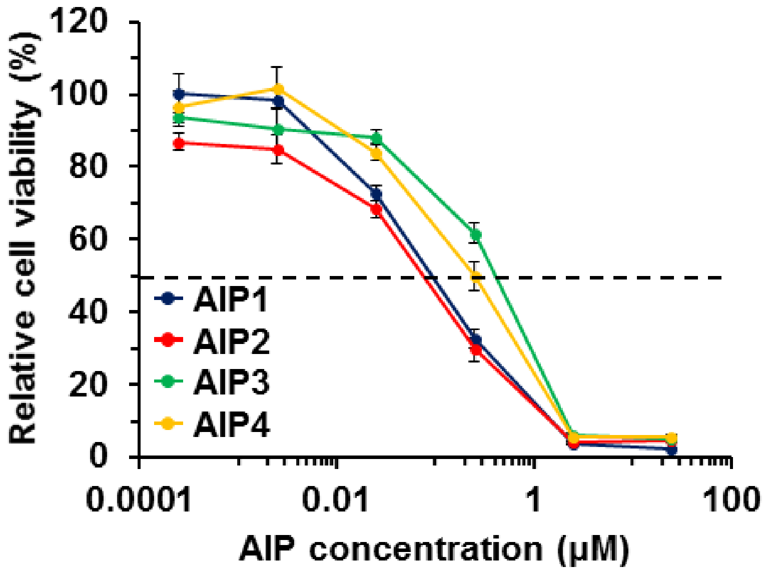

Figure S16. Relative cell viability of AIP series in NCI-H460 at different concentrations (25, 2.5, 0.25, 0.025, 0.0025, 0.00025 μM), as evaluated by MTT assay (*n*=4, S.D.).

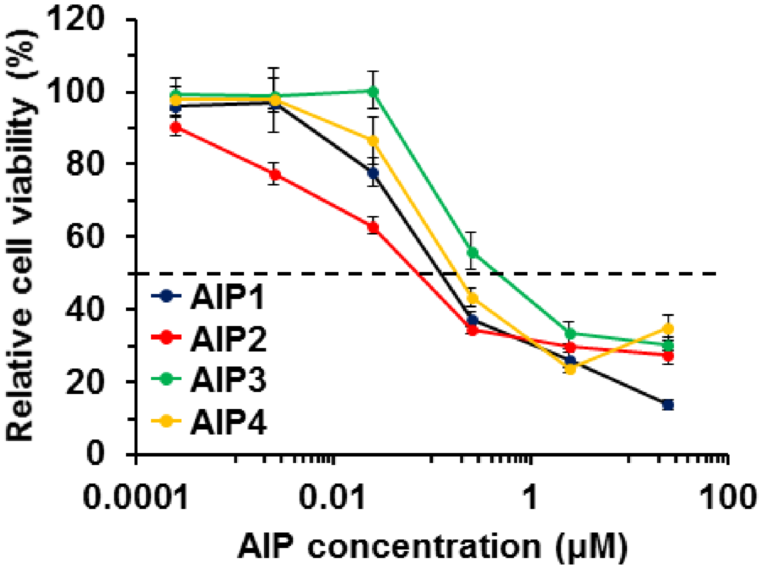

Figure S17. Relative cell viability of AIP series in MDA-MB-231 at different concentrations (25, 2.5, 0.25, 0.025, 0.0025, 0.00025 μM), as evaluated by MTT assay (*n*=4, S.D.)

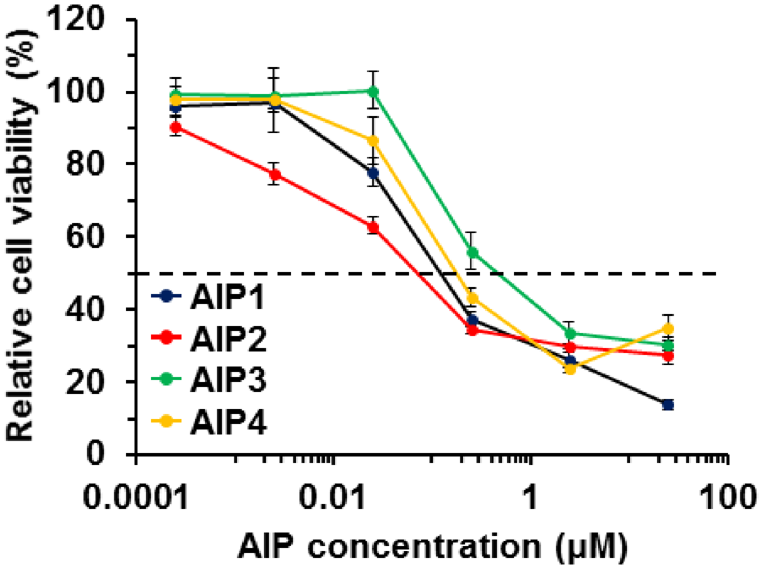

Figure S18. Relative cell viability of AIP series in HT-1080 at different concentrations (25, 2.5, 0.25, 0.025, 0.0025, 0.00025 μM), as evaluated by MTT assay (n=4, S.D.)

Entry	AIP1	AIP2	AIP3	AIP4
**NCI-H460 IC_{50} (μM)**	0.094	0.050	0.185	0.280
**MDA-MB-231 IC_{50} (μM)**	0.209	0.216	1.212	0.559
**HT-1080 IC_{50} (μM)**	0.282	0.287	0.354	0.527

Supplementary Table 1. Summarized IC50 value of AIP series obtained by the logarithm regression.

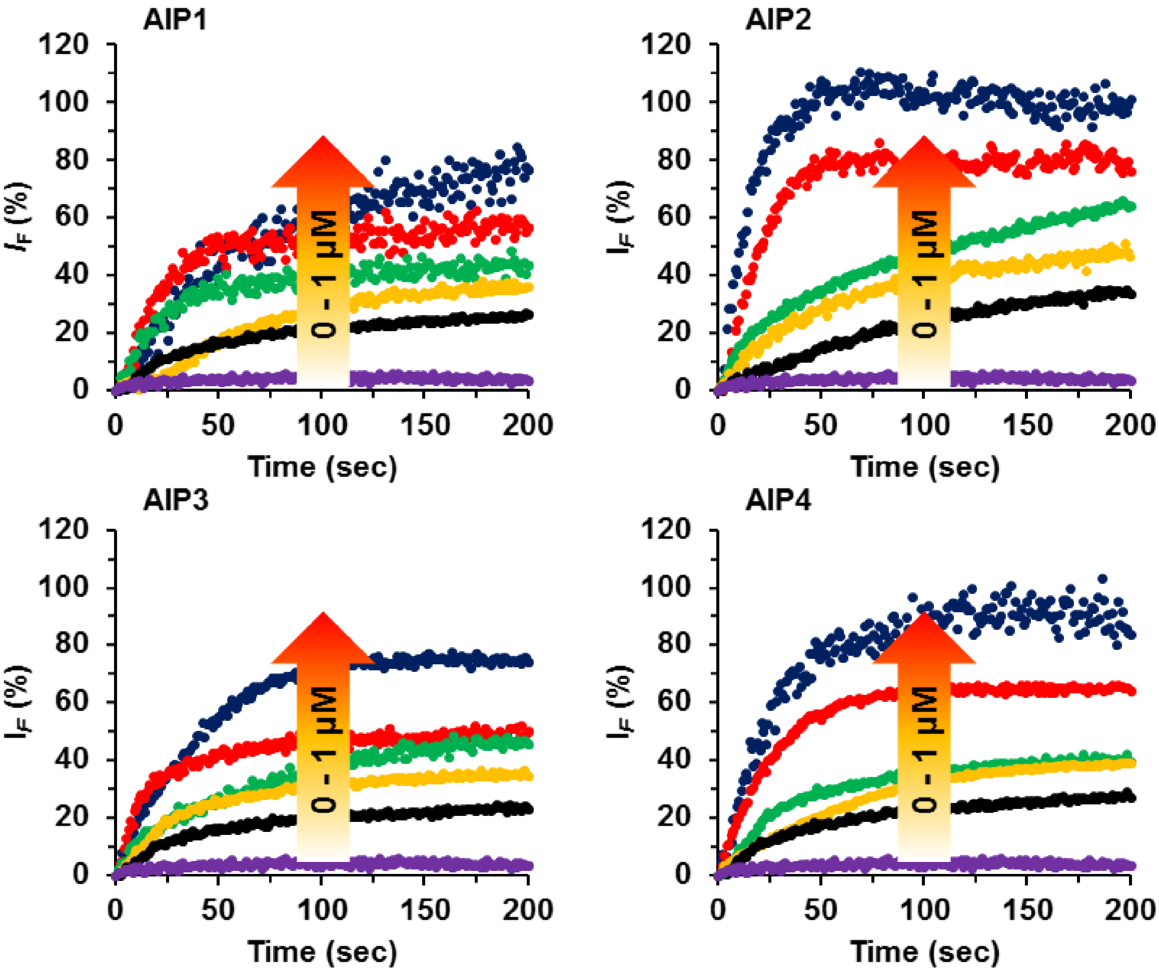

Figure S19. Concentration-dependent HPTS assay performed by the treatment of AIPs (1, 0.75, 0.5, 0.25, 0.125 μM).

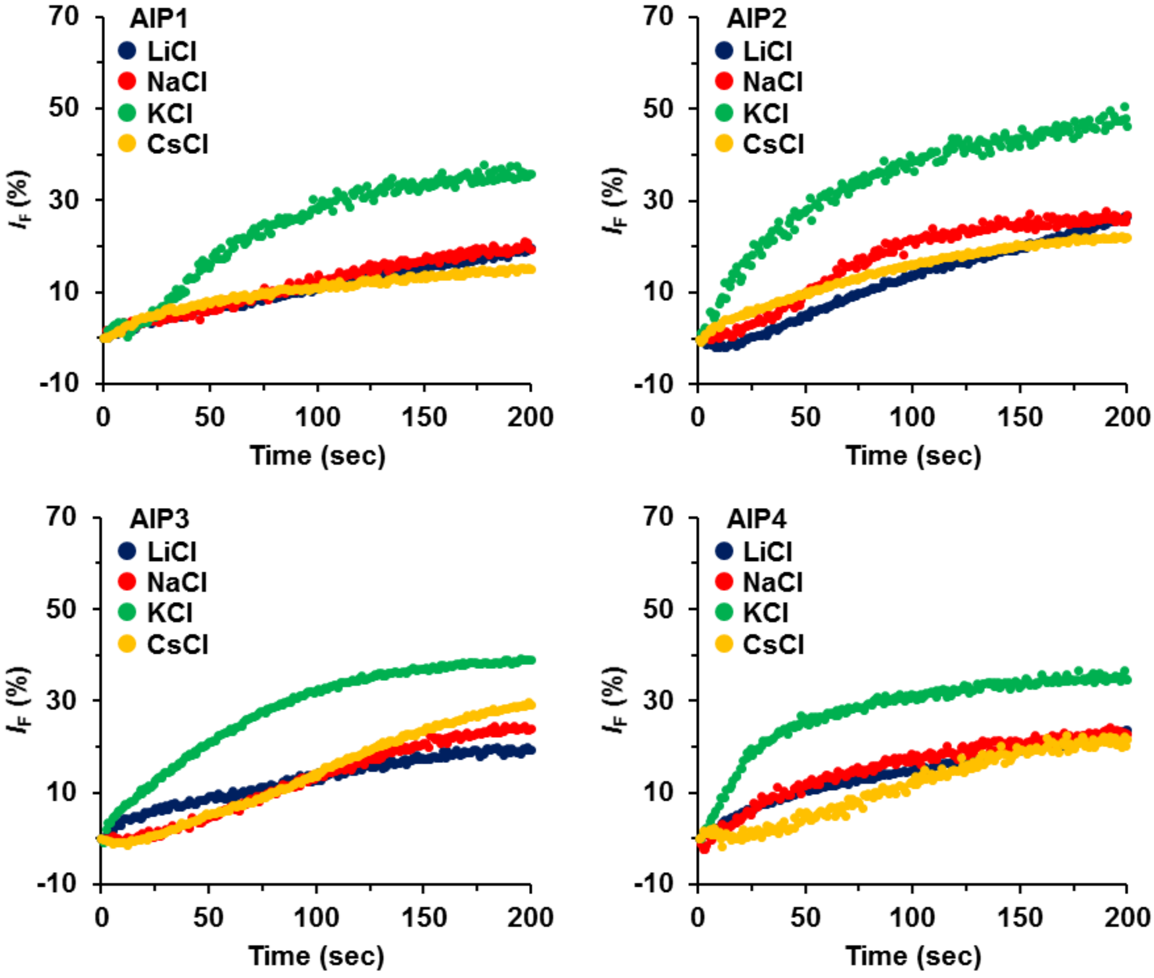

Figure S20. Ion transport activity of AIP series (0.25 μM) across HPTS-loaded LUVs determined by varying cations (LiCl, NaCl, KCl, and CsCl).

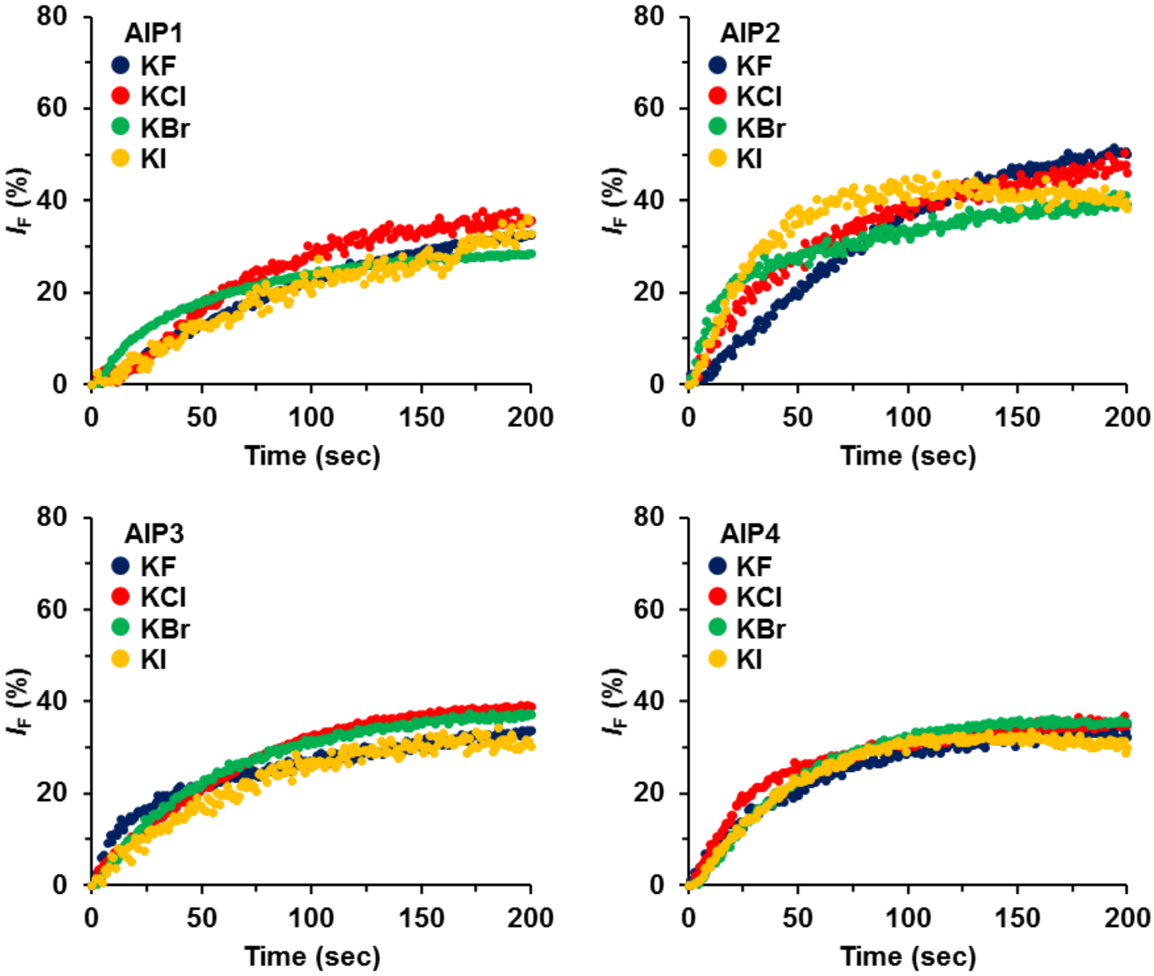

Figure S21. Ion transport activity of AIP series (0.25 μM) across HPTS-loaded LUVs determined by varying anions (KF, KCl, KBr, and KI).

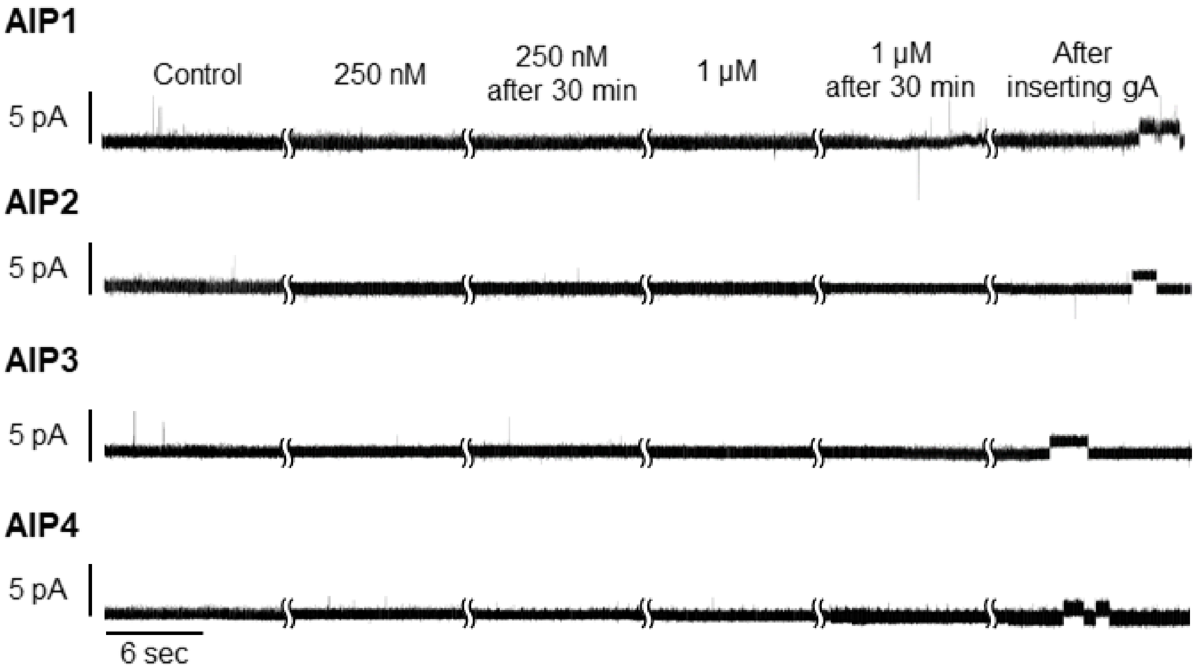

Figure S22. Current trace represents events prior to and after addition of AIPs.

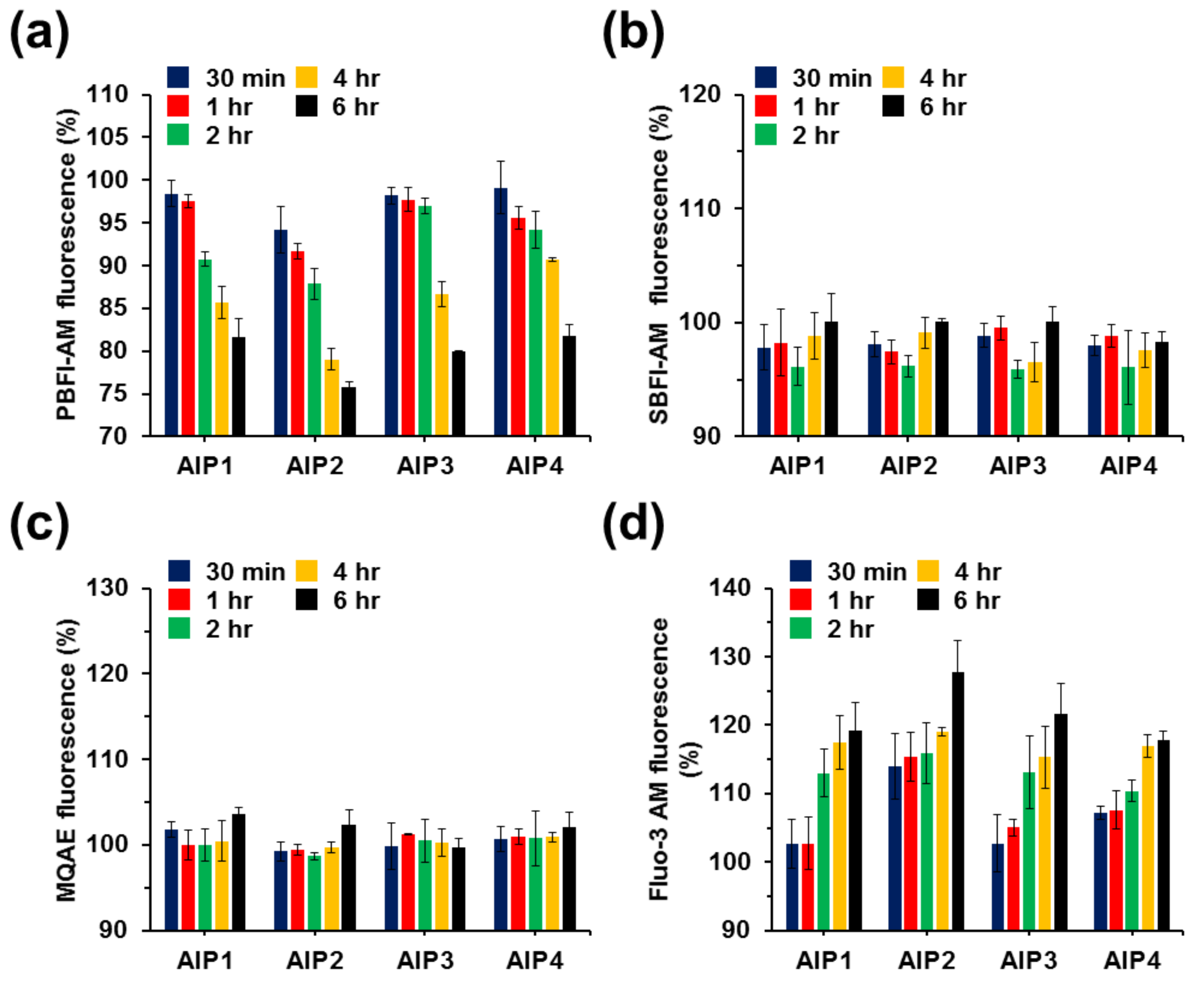

Figure S23. Time-dependent intracellular ion concentrations by the treatment of AIPs (0.75 μM). (a) potassium, (b) sodium, (c) chloride and (d) calcium.

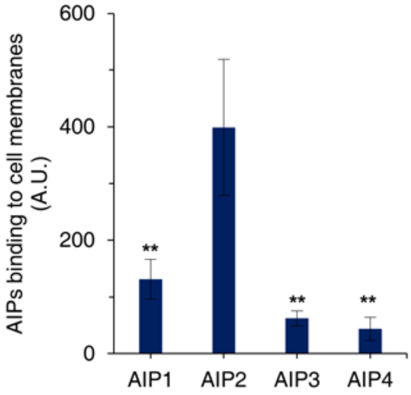

Figure S24. AIPs binding to cell membemranes evaluated by flow cytometry. (n=3, S.D.) $**p < 0.01$ (compared to AIP2) (*t*-test).

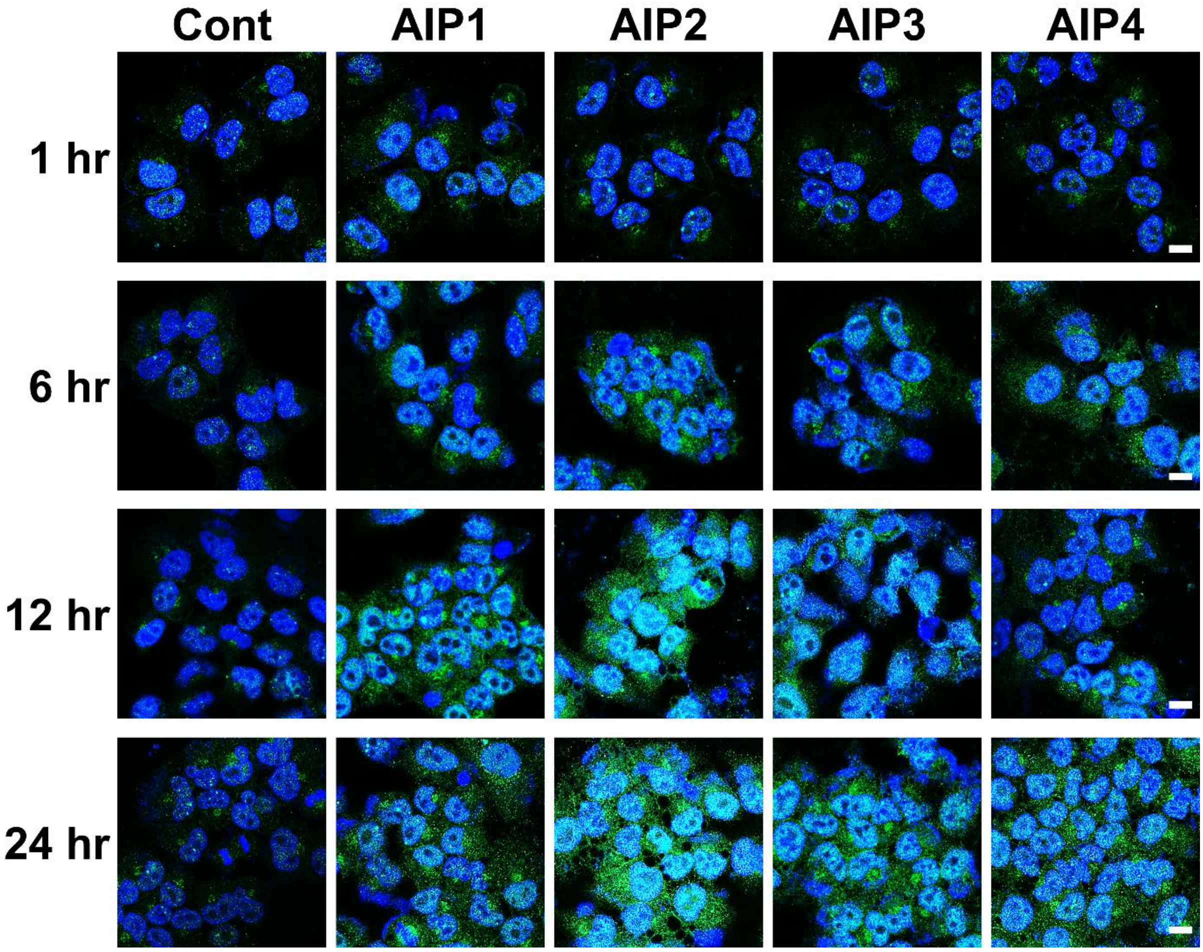

Figure S25. Immunofluorescence images of CHOP taken by CLSM after the treatment of none and AIPs for 1, 6, 12, and 24 hr. (Green fluorescence; CHOP, blue fluorescence; DAPI, Scale bar; 10 μm)

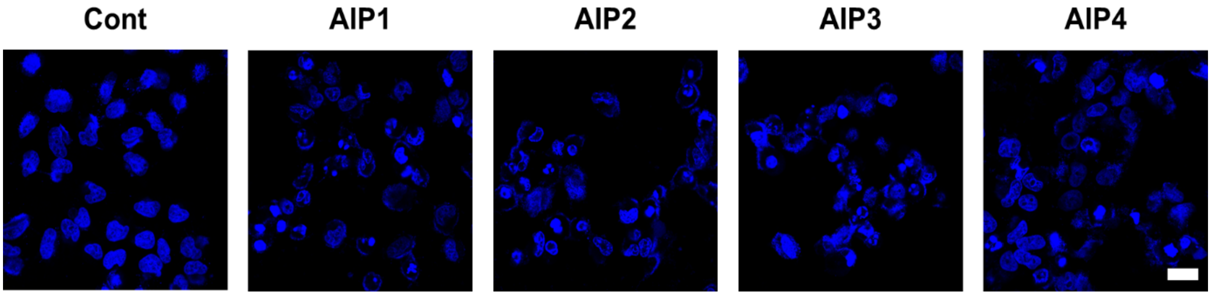

Figure S26. Visualization of nuclear fragmentation by the treatment of AIPs (0.25 μM) for 12 hr. Nuclei were stained using DAPI (Scale bar; 20 μm).

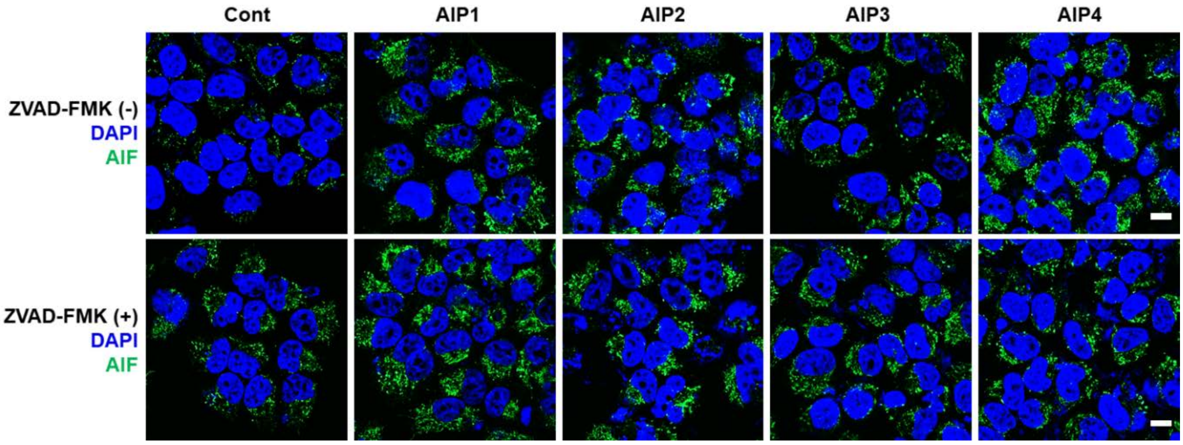

Figure S27. Immunofluorescence images of AIF with or without the pretreatment of ZVAD-FMK (40 μM) before the treatment of AIPs AIPs (0.25 μM) for 1 day (Scale bar; 10 μm).

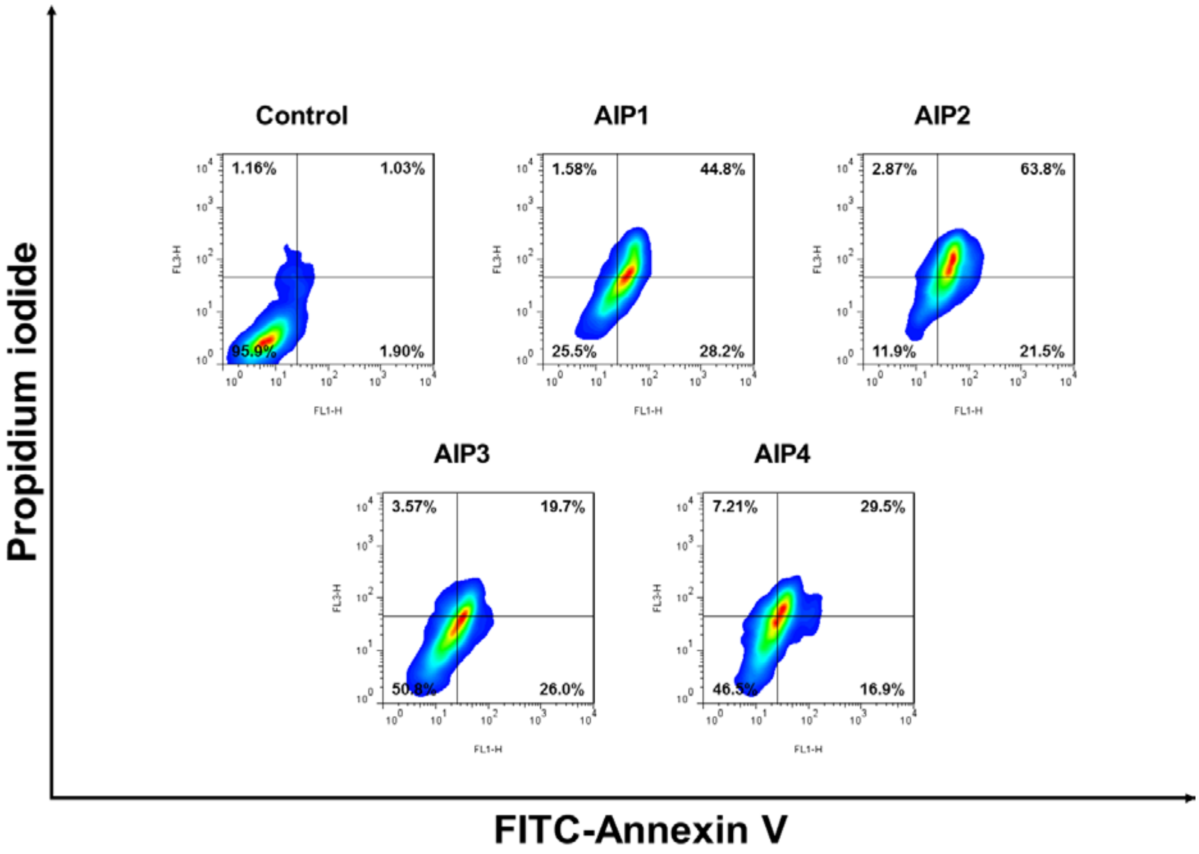

Figure S28. FACS analysis for apoptosis observation using FITC-Annexin V and propidium iodide staining in NCI-H460 cells on the treatment of AIP series (0.25 μM).

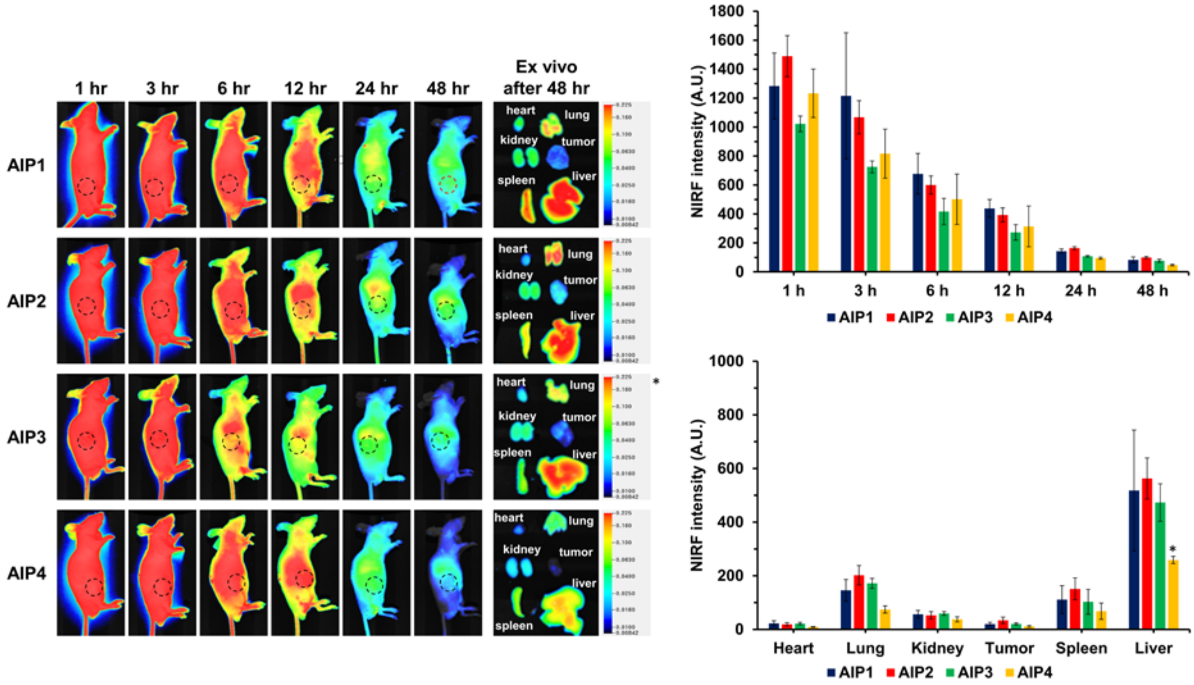

Figure S29. (a) Time-dependent NIRF imaging treated with IR800CW-tagged AIPs. NIRF detection of excised organs and tumor after 48 h. (b) Time-dependent NIRF intensity of IR800CW-tagged AIPs localized in tumor (n=3, S.D.). (c) NIRF intensity of the excised organs and tumor after 48 h (n=3, S.D.). *$p < 0.05$(compared to AIP2) (*t*-test).

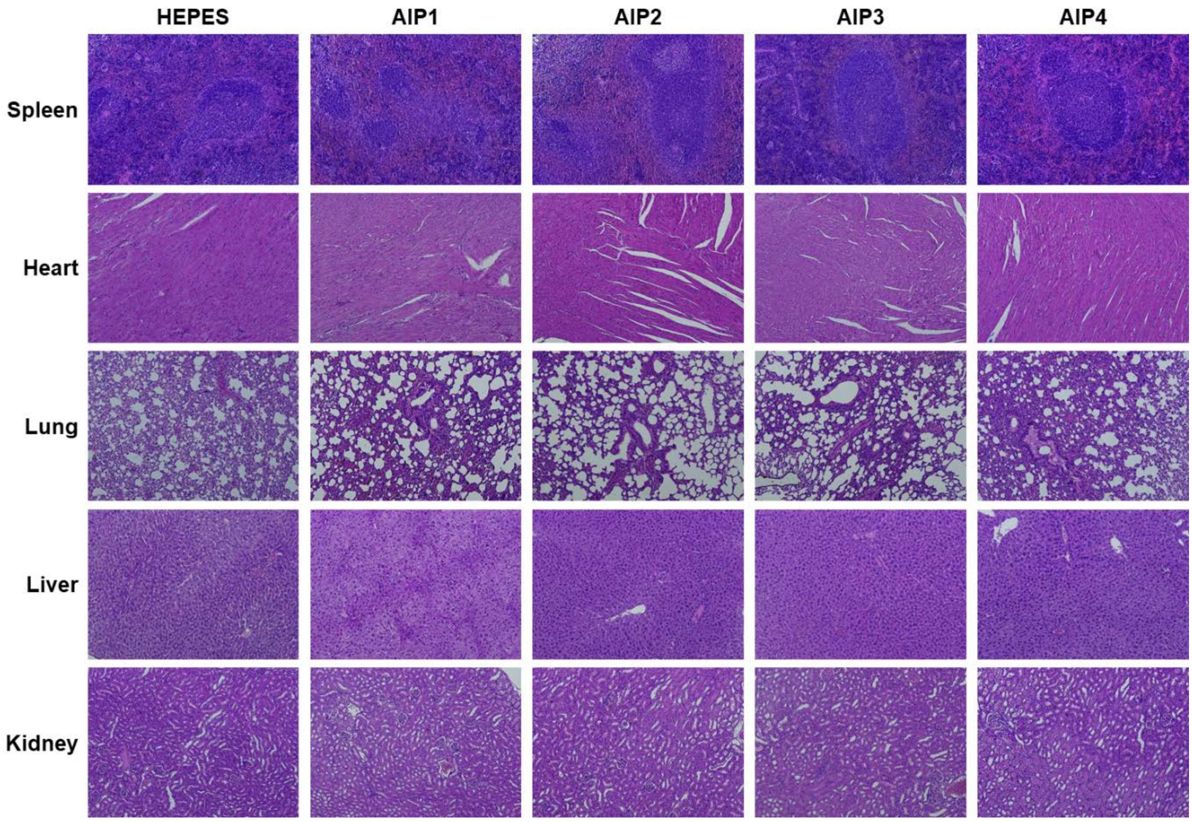

Figure S30. H&E images of harvested organs (Spleen, heart, lung, liver, and kidney) after all the mice were sacrificed. Magnification was 400 X.

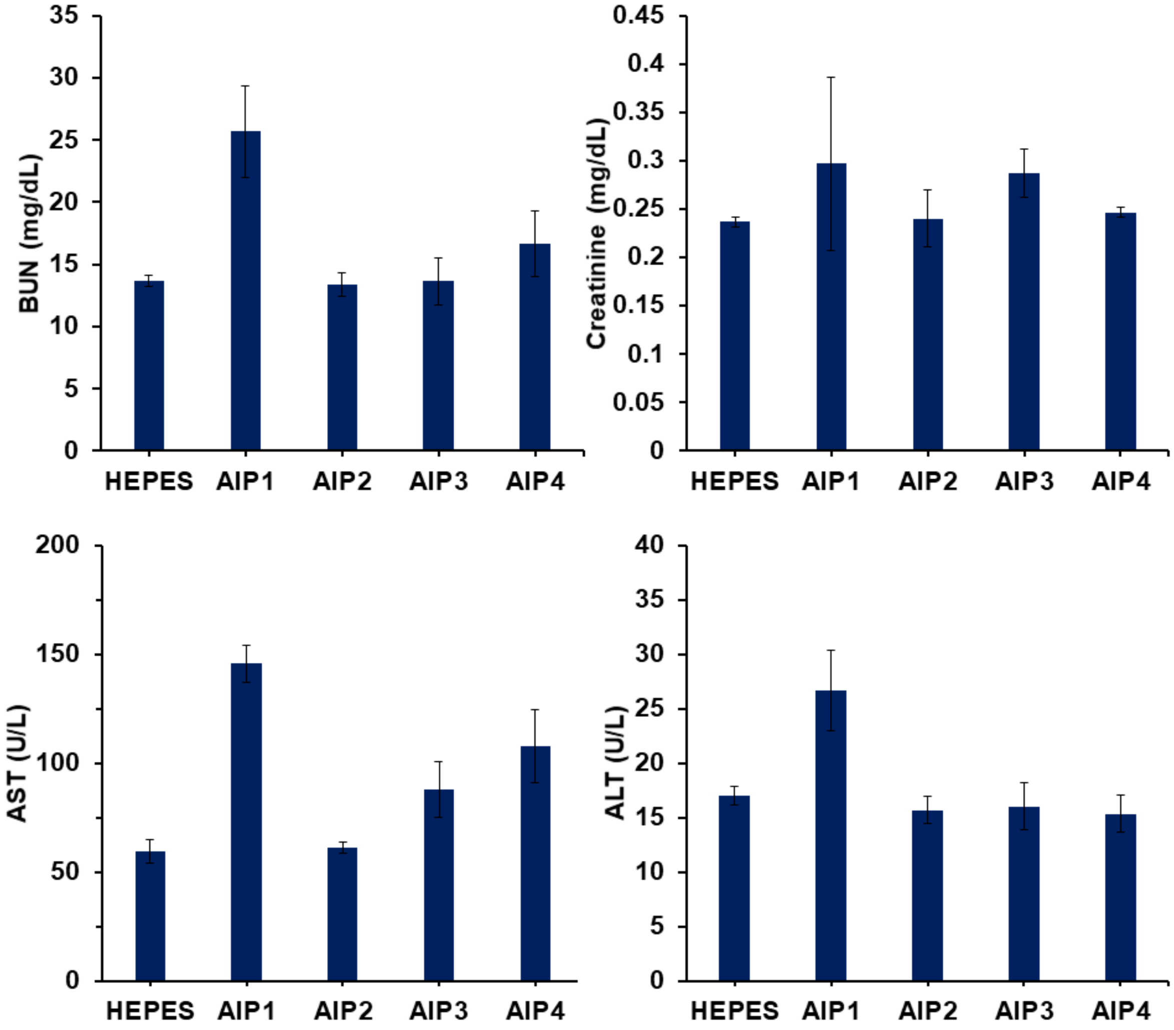

Figure S31. The levels of BUN, AST, ALT, and creatinine in blood samples collected from each mouse group. (*n*=3, S.D.) Normal range (BUN: 8-33 mg/dL, creatinine: 0.2-0.9 mg/dL, AST: 54-298 U/L, ALT: 17-77 U/L).

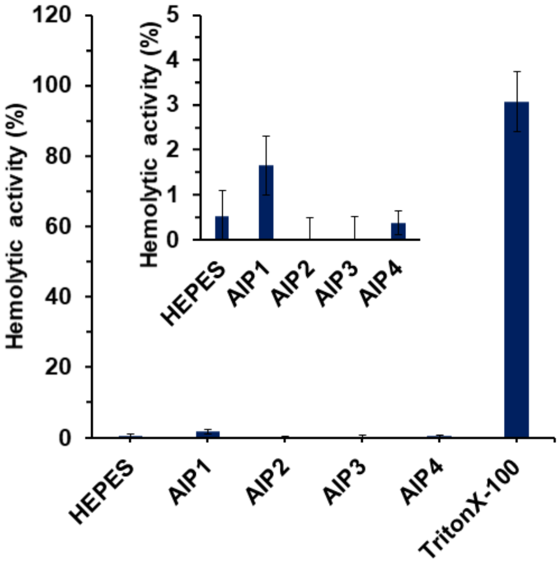

Figure S32. Hemolytic activity of HEPES, AIPs, and TritonX-100 using mouse RBCs. (*n*=3, S.D.)

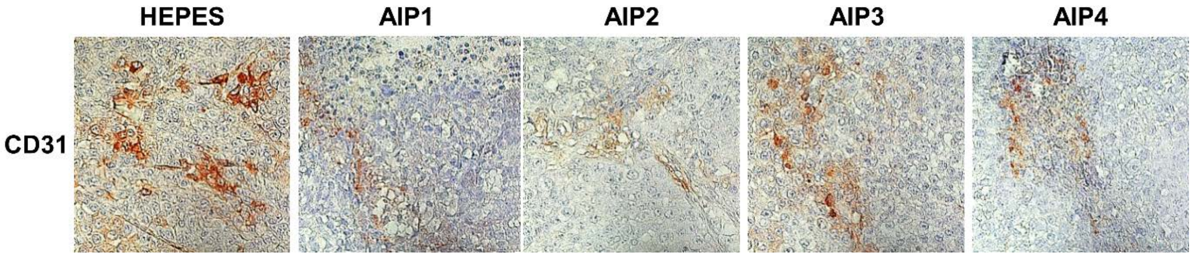

Figure S33. Immunohistochemical assays of the harvested tumor sections for CD31 used as a blood vessel marker. All images were taken by an optical microscope (magnification: 400 X).

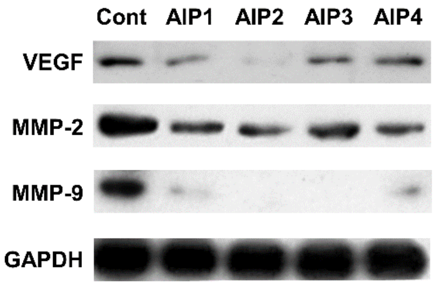

Figure S34. Demonstration of angiogenesis-related proteins in vitro (VEGF: vascular endothelial growth factor, MMP-2: matrix metalloproteinase-2, MMP-9: matrix metalloproteinase-9*)* via immunoblotting. GADPH was used as a loading control.

References

1 Saha, T., Gautam, A., Mukherjee, A., Lahiri, M. & Talukdar, P. Chloride Transport through Supramolecular Barrel-Rosette Ion Channels: Lipophilic Control and Apoptosis-Inducing Activity. *J Am Chem Soc* **138**, 16443-16451, doi:10.1021/jacs.6b10379 (2016).

2 Ryu, H. *et al.* Investigation of Ion Channel Activities of Gramicidin A in the Presence of Ionic Liquids Using Model Cell Membranes. *Scientific reports* **5**, 11935 (2015).

3 Lee, E.-B. *et al.* Synthetic anion transporters that bear a terminal ethynyl group. *Chemical Communications* **51**, 9339-9342 (2015).

4 Mueller, P., Rudin, D. O., Ti Tien, H. & Wescott, W. C. Reconstitution of Cell Membrane Structure in vitro and its Transformation into an Excitable System. *Nature* **194**, 979-980 (1962).

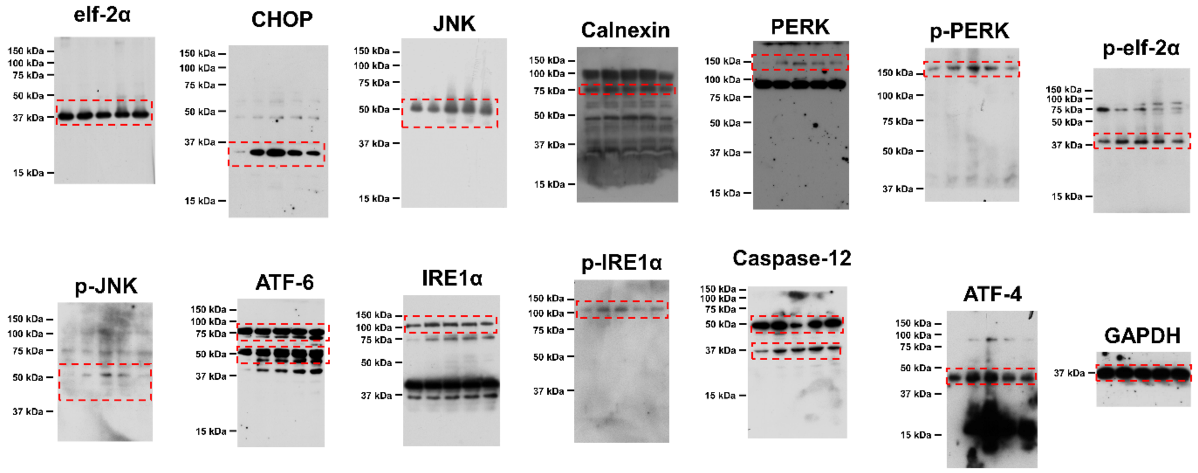

Uncropped western blot images for ER stress *in vitro* (lane 1: Cont, Lane 2: AIP1, Lane 3: AIP2, Lane4: AIP4, Lane 5: AIP4).

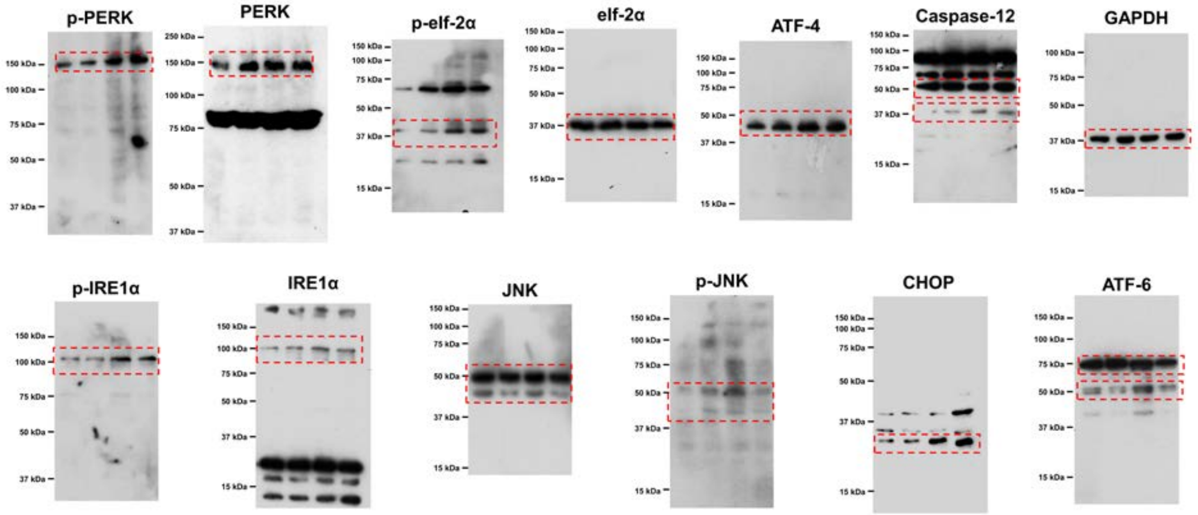

Uncropped western blot images for ER stress *in vitro* with or without ZVAD-FMK (lane 1: Cont, Lane 2: Cont with ZVAD-FMK, Lane 3: AIP2, Lane4: AIP2 with ZVAD-FMK).

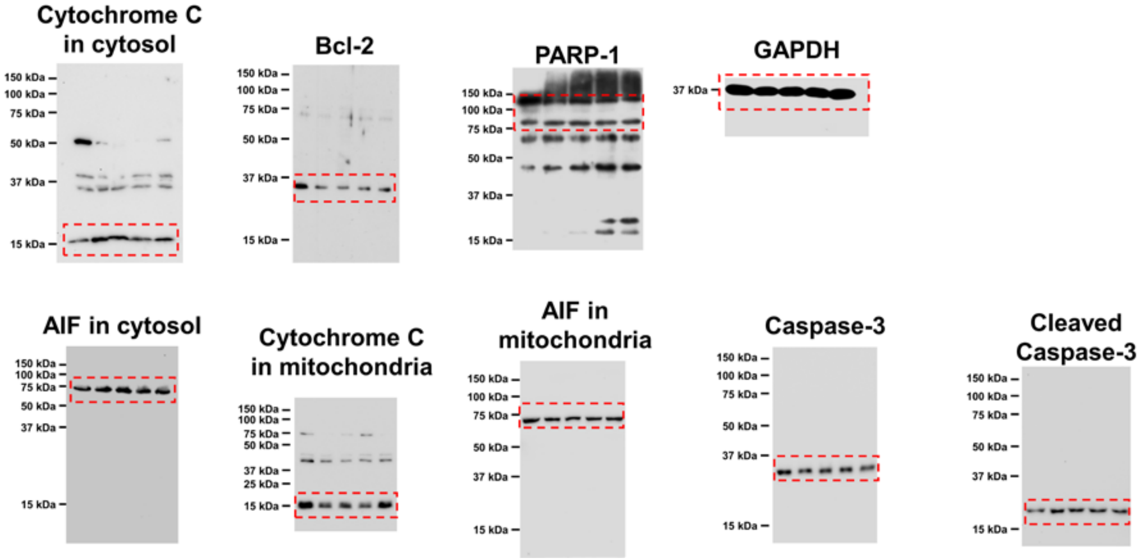

Uncropped western blot images for apoptosis *in vitro* (lane 1: Cont, Lane 2: AIP1, Lane 3: AIP2, Lane4: AIP4, Lane 5: AIP4).

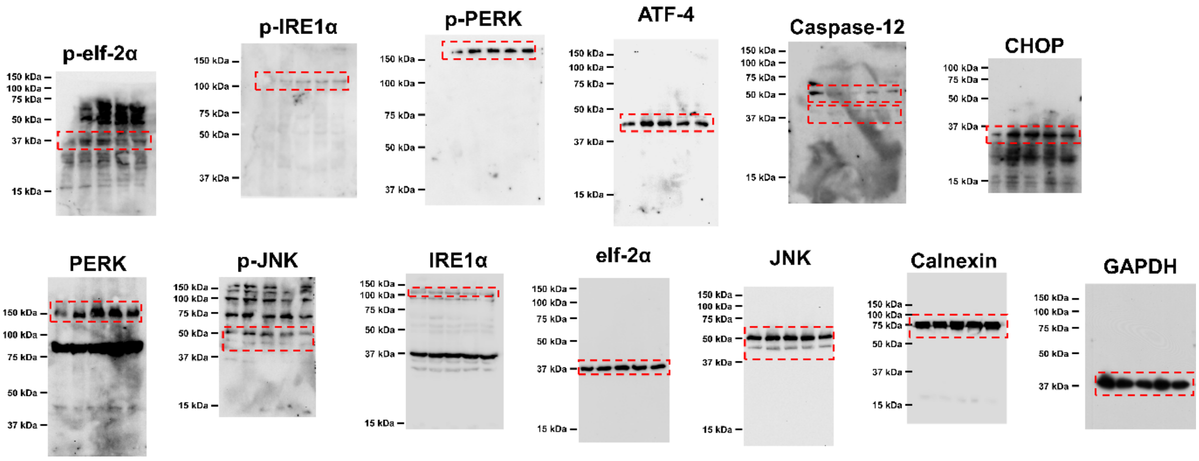

Uncropped western blot images for ER stress *in vivo* (lane 1: HEPES, Lane 2: AIP1, Lane 3: AIP2, Lane4: AIP4, Lane 5: AIP4).

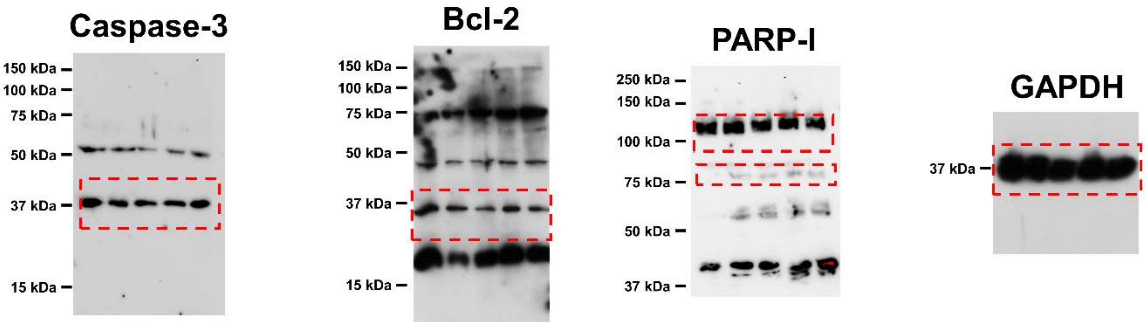

Uncropped western blot images for apoptosis *in vivo* (lane 1: HEPES, Lane 2: AIP1, Lane 3: AIP2, Lane4: AIP4, Lane 5: AIP4).
